# Fermentation of Green Tea with 2% *Aquilariae lignum* Increases the Anti-Diabetic Activity of Green Tea Aqueous Extracts in the High Fat-Fed Mouse

**DOI:** 10.3390/nu7115447

**Published:** 2015-11-03

**Authors:** Ji Eun Lee, Su Jin Kang, Seong Hun Choi, Chang Hyun Song, Young Joon Lee, Sae Kwang Ku

**Affiliations:** 1 The Medical Research Center for Globalization of Herbal Medicine, Daegu Haany University, Gyeongsan, Gyeongsangbuk-Do 38610, Korea; jelee910@naver.com (J.E.L.); vegonia1@hanmail.net (S.J.K.); dvmsong@hotmail.com (C.H.S.); 2 Department of Histology and Anatomy, College of Korean Medicine, Daegu Haany University, 1, Hannydaero, Gyeongsan, Gyeongsangbuk-Do 38610, Korea; ck0190@hanmail.net; 3 Department of Preventive medicine, College of Korean Medicine, Daegu Haany University, 1, Hannydaero, Gyeongsan, Gyeongsangbuk-Do 38610, Korea

**Keywords:** high fat diet, mouse, obese, diabetes, *Aquilariae lignum*, fermented green tea, metformin, simvastatin, hepatic glucose-regulating enzyme

## Abstract

Anti-diabetic effects on the metabolomic differences between green tea (GT) and *Aquilariae lignum*-fermented green tea (fGT) were investigated in the high fat-fed mouse. To prove the differences, hypoglycemic (blood glucose, insulin and glycated hemoglobin levels, pancreas weights and histopathological-immunohistochemistrical analysis of pancreas–insulin/glucagon cells), hepato- and nephron-protective (the changes in liver and kidney weight, histopathology of liver and kidney, serum aminotransferases (AST and ALT) levels, blood urea nitrogen, and serum creatinine levels), and hypolipidemic (the changes of serum total cholesterol, triglyceride, low- and high-density lipoprotein levels with fecal TC and TG contents) effects were evaluated. In addition, liver lipid peroxidation, the glutathione contents, and catalase and superoxide dismutase activities were measured according to the hepatic glucose-regulating enzyme activities of glucokinase (GK), glucose-6-phosphatase (G6pase) and phosphoenolpyruvate carboxykinase (PEPCK) for action mechanisms. As a result, fGT showed a stronger hypoglycemic, hepato- and nephron-protective, hypolipidemic, and anti-oxidant effect than GT in high fat-fed mice. In addition, fGT-treated mice exerted more favorable inhibitory activities against GK, G6pase, PERCK activities as compared to GT-treated mice. Taken together, fGT fermented with *Aquilariae lignum*, 1:49 (2%; g/g) has a stronger effect compared with GT. Therefore, fGT has the potential to increase bioactivity against type 2 diabetics.

## 1. Introduction

Obesity contributes to the etiologies of a variety of comorbid conditions, such as cardiovascular disease, hypertension, and type II diabetes [1]. In addition to storing lipid for energy, adipose secretes a variety of adipokines, many of which affect metabolism and inflammation in adipose and non-adipose tissues. Modulation of the endocrine functions of adipose tissue can contribute to a chronic state of inflammation, which leads to the pathogenesis of associated disorders, specifically insulin resistance [2]. One of the critical determinants for the development of this obesity may be an increase in the regional distribution of body fat, *i.e.*, abdominal obesity. The latter often shows clustering of atherogenic risk factors [3], *i.e.*, hypertension, dyslipidemia, alterations in coagulation and inflammatory cytokine profiles, and hyperinsulinemic insulin resistance. As a consequence, there is an expected increase in morbidity and mortality of cardiovascular disease [4].

Currently available pharmacological agents for metabolic syndrome, however, have a number of limitations, such as various adverse effects and high rates of secondary failure [5]. Due to these factors, metabolic syndrome patients and healthcare professionals are increasingly considering complementary and alternative approaches. Since control of postprandial hyperglycemia and inhibition of oxidative stress are suggested to be important in the treatment of diabetes [6], many efforts had been made to search for effective and safe α-glucosidase inhibitors and antioxidants from natural materials to develop a physiological functional food or lead compounds for curing diabetes [7,8]. Among them, metformin is an oral antidiabetic drug in the biguanide class. It is the first-line drug of choice for the treatment of type 2 diabetes, in particular, in overweight and obese people and those with normal kidney function [9]. It is the only antidiabetic drug that has been conclusively shown to prevent the cardiovascular complications of diabetes, and also metformin has been shown to have regulatory effects on pancreatic zymogen release [10] and hepatic glucose-regulating enzyme activities [11,12].

In recent years, fermented herbs have been highlighted as a new source of medicinal ingredients or pharmaceutics, because the bioactivity of natural herbs is increased by various fermentation techniques through their biotransformation or probiotic effects [10,13,14,15]. Moreover, fermentation of herbs using a variety of edible microorganisms seems to further enhance the pharmacological efficacy of parent herbs [10,13,14,15]. Tea (*Camellia sinensis* L.) is the second most consumed beverage in the world next to water. Tea was found to demonstrate bioactivities including antioxidant activities [16], improved immune response [17], and anti-atherosclerosis [18], antihypertension [19], anti-infectious diseases [20], and antidiabetic properties [21]. The chemical compositions of the tea in different forms were different and induced the change in bioactivities [6,22]. *Aquilariae lignum* is the stem part of *Aquilaria agallocha* Roxb (*Thymelaceae*) and contains essential oils. The chemical composition of *Aquilariae lignum* includes benzylacetone, *p*-methoxybenzylacetone, hydrocinnamic acid, agarospirol, agarofuran and dihydroagarofuran [23,24]. *Aquilariae lignum* has been traditionally used in aroma-therapeutics for various purposes, and anti-allergic [25], analgesic [26] and anxiolytic [27] effects have been reported. In our previous study [10], aqueous extracts of fermented green tea with *Aquilariae lignum* (fGT) effectively inhibit diabetes and related complications—diabetic hyperlipidemia, hepatopathies, nephropathies and obesity in db/db mice—more favorably than those of aqueous green tea extract (GT).

Excessive intake of fatty acids leads to an accumulation of triglyceride in many tissues, particularly in the fat tissue, in which lipolysis is increased. The increased circulation of fatty acids, associated with rising lipolysis in adipocytes with insulin resistance, results in a plethora of fatty acids to non-adipose tissues, such as muscle, pancreas and liver. In individuals with insulin resistance, increased levels of tissue fatty acid-binding and transport proteins in adipose and non-adipose tissues facilitate the uptake processes. The exaggerated availability of free fatty acid (FFA) and deposition in muscle induces a negative loop in insulin-mediated muscle insulin signaling and glucose utilization. In the pancreas, prolonged exposure to FFA might cause impairment of insulin release through the mechanism of lipotoxicity [28]. In the liver, high FFA concentration contribute to resistance to the action of insulin by enhancing glucose output from liver [29]. The accumulation of triglyceride in liver by high fatty acids also brings about non-alcoholic fatty liver disease (NAFLD). NAFLD does damage to liver, which is the main organ of glucose metabolism, such as steatosis, steatohepatitis, and hepatocellular necrosis to fibrosis [30]. The balance between hepatic lipogenesis and lipolysis is important for improving insulin resistance and NAFLD. These are generally characteristic features of metabolic syndrome [31].

In this study, we intended to confirm or observe the real pharmacological activities of fGT in mild diabetic obese mice and high fat diet (HFD; 45 kcal% fat) supplied mice [14,32,33,34] as compared with parent GT. Metformin, a representative anti-diabetic drug for type II diabetes [35,36], at a dose level of 250 mg/kg [10,14,15], and simvastatin, a lipid lowering medication [37,38] which has been used for the treatment of dyslipidemia and the prevention of cardiovascular disease in diabetes [39,40] at a dose level of 10 mg/kg [14,15], were used as potent reference drugs.

## 2. Experimental Section

### 2.1. Animals and Husbandry

Female SPF/VAF CrljOri:CD1 [ICR] mice (6 weeks old upon receipt; OrientBio, Seungnam, Korea) were used after acclimatization for 7 days. Four to five animals were placed in each polycarbonate cage in a temperature (20 °C–25 °C) and humidity (40%–45%) controlled room. Light: dark cycle was 12 h:12 h, and standard rodent chow (Samyang, Seoul, Korea) and water were supplied as free to access. Animals adapted to HFD were selected after a 1-week adaption period as eight groups (eight mice in each groups) based on the body weights. All laboratory animals were treated according to the national regulations of the usage and welfare of laboratory animals, and approved by the Institutional Animal Care and Use Committee in Daegu Haany University (Gyeongsan, Gyeongbuk, Korea) prior to animal experiment (Approval No. DHU2014-066).

### 2.2. Preparation and Administration of Test Substances

Light brown solution of fGT and greenish brown solution of GT were prepared by ChuiWoon HyangDang (Seongju, Korea) according to our previous study [10]. The process for making fGT was as follows: Briefly, mixtures of dried green tea leaves and *Aquilariae lignum* powder (49:1, g/g) were wet-fermented for 12 h at 60 °C, and then steamed for 30 s at 100 °C after being dried for 1 week at 15 °C. The steamed mixtures were cooled and additionally dried at 15 °C for 3 days. Each of fGT or dried GT (28 g) were boiled at 100 °C for 6 h and then cooled for an additional 6 h in 1 L of pure water, respectively. Aqueous solutions were completely lyophilized (Operon FDB-5503, Kimpo, Korea). Total 5.40 g of fGT (yield = 19.29%) and 7.28 g of GT (yield = 26.00%) were acquired, and used in this study. Lyophilized fGT and GT aqueous extracts were stored at −20 °C in a refrigerator to protect them from light and humidity until used. Metformin hydrochloride (Wako Pure Chemical, Osaka, Japan) and simvastatin (Bicon Limited, Bamgalore, India) were used as reference recommendation drugs. Each of fGT 400, 200 and 100 mg/kg, simvastatin 10 mg/kg, metformin 250 mg/kg or GT 400 mg/kg were orally administered, dissolved in distilled water, once a day for 84 days from 14 days of acclimatization in a volume of 10 mL/kg, respectively. In intact vehicle and HFD control mice, equal volumes of distilled water were also orally administered, instead of test substances, respectively. The dosages of GT (400 mg/kg) and fGT (400, 200 and 100 mg/kg) were selected based on our previous experiment in db/db mice [10], respectively. The dose levels of metformin, 250 mg/kg [10,14,15] and simvastatin 10 mg/kg [14,15] were also selected as per our previous animal studies, respectively.

### 2.3. HFD Supply

Animals were supplied HFD (Research Diet, New Brunswick, NJ, USA) free to access listed in Table 1 after 7 days of acclimatization. In intact control, normal pellet diets (Superfeed Co., Wonju, Korea) were supplied as free to access instead of HFD.

### 2.4. Changes in Body Weight

Changes in body weight were measured at 8 days (immediately before start of HFD supply) and 1 day before initiation of administration, and at initial administration day, and then weekly until termination using an automatic electronic balance (Precisa Instrument, Dietikon, Switzerland). At initiation of administration and at termination, all experimental animals were overnight fasted (but not for water; about 12 h) to reduce the differences from feeding. In addition, body weight gains were additionally calculated during adaption and administration periods, using the following equation: during adaption periods (7 days; from Day −8 to Day 0 of test article-administration) = (body weights at initiation of administration − body weights at initiation of HFD supply); during administration periods (84 days; from Day 0 to Day 84 of test article administration) = (body weight at a termination − body weight at initiation of administration).

**Table 1:** Formulas for normal and high fat diets used in this study.

Compositions	Normal Pellet Diets (g/kg)	High Fat Diets (g/kg)*
Ingredient		
Casein	200	200
l-Cystein	3	3
Corn starch	150	72.8
Sucrose	500	172.8
Cellulose	50	50
Soybean Oil	50	25
Lard	0	177.5
Mineral mixture	35	35
Vitamin mixture	10	10
Choline bitartrate	2	2
Energy (kcal/g)	4.00	4.73
Protein (% kcal)	20	20
Carbohydrate (% kcal)	64	35
Fat (% kcal)	16	45

* 45 kcal% fat pellet diets (D12451; Research Diet, New Brunswick, NJ, USA) were used as high fat diet (HFD) and normal rodents pellet diets (Superfeed Co., Wonju, Korea) were used as normal fat pellet diet.

### 2.5. Mean Daily Food Consumption and Mean Daily Energy Intake Measurements

Diets (150 g in each individual cage) were supplied, and remaining amounts of supplied diets were measured at 24 h after using an automatic electronic balance (Precisa Instrument, Dietikon, Switzerland). This was divided by the number of reared animals in the same cage, to obtain the individual mean daily food consumption of mice (g/day/mice). Mean daily energy intakes of mice were calculated by multiplying the calories of each diet by mean daily food consumption, and then by calculating the calories per gram of body weight. These measurements were made once a week during 84 days of administration according to our previous reports [14,15].

### 2.6. Measurement of Body Fat Distribution: Total and Abdominal Fat Mass (%)

The mean fat densities in the total body and abdominal cavity regions of each mouse were detected by *in live* DEXA (InAlyzer, Medikors, Seungnam, Korea) once at the end of the 84 days of continuous treatment of test substances.

### 2.7. Measurement of Blood Glucose Level

At the end of the 84 days continuous treatment, blood was collected from *vena cava*, and deposited into a NaF glucose vacuum tube (Becton Dickinson, Franklin Lakes, NJ, USA) and plasma was separated. Blood glucose levels were measured using an automated blood analyzer (Toshiba 200 FR, Toshiba, Tokyo, Japan).

### 2.8. Serum Biochemistry

Collected blood from *vena cava* at 84 days after initial test substance treatment was deposited into clotting activated serum tubes, and centrifuged at 15,000 rpm for 10 min at room temperature in order to separate the serum for aspartate aminotransferase (AST), alanine aminotransferase (ALT), blood urea nitrogen (BUN), creatinine, total cholesterol (TC), triglyceride (TG), low density lipoprotein (LDL) and high density lipoprotein (HDL) measurements. Serum AST, ALT, BUN, creatinine, TC and TG levels were measured using an automated blood analyzer (Hemagen Analyst, Hemagen Diagnostic, Columbia, MD, USA), and serum HDL and LDL were also detected by type using an automated blood analyzer (AU400, Olympus, Tokyo, Japan), respectively.

### 2.9. Measurement of Serum Insulin and Blood HbA1c Level

Blood HbA1c and serum insulin levels were determined using a HbA1c Measuring System (Infopia, Anyang, Korea) and an ELISA kit (Alpco Diagnostics, Windham, NH, USA), according to previously established methods [34,41].

### 2.10. Organ Weight Measurements

At termination, the weights of liver, pancreas, left kidney, left periovarian fat pads and abdominal wall deposited fat pads attached to the muscularis quadratus lumborum were measured at g levels, individually, and to reduce the differences from individual body weights, the relative weights (% of body weights) were also calculated using body weight at sacrifice and absolute weight using the following equation: Relative organ weights (%) = ((Absolute organ weights/Body weight at sacrifice) × 100), according to our previously established methods with some modifications [10,14,15].

### 2.11. Measurement of Lipid Compositions in the Feces

Lipid was extracted from feces collected at 8 h after last test substance administration, according to the method of Folch *et al.* [42]. The concentrations of fecal TC and TG were measured enzymatically using a commercial kit (Asan Pharmaceutical Co. Seoul, Korea) based on a modification of the lipase–glycerol phosphate oxidase method [10,43].

### 2.12. Liver Lipid Peroxidation and Antioxidant Defense Systems

After measurements of organ weights, the malondialdehyde (MDA) and glutathione (GSH) contents and catalase (CAT) and superoxide dismutase (SOD) enzyme activities in mouse hepatic tissues were assessed, respectively. The liver was homogenized in ice-cold 0.01 M Tris-HCl (pH 7.4), and centrifuged, at 12,000× *g* for 15 min as described elsewhere [44]. The lipid peroxidation was determined by estimating MDA using the thiobarbituric acid test at absorbance of 525 nm, as nM of MDA/mg tissue [45]. Total protein was measured using bovine serum albumin (Invitrogen, Carlsbad, CA, USA) as internal standard [46]. The homogenates were mixed with 0.1 mL of 25% trichloroacetic acid (Merck, San Francisco, CA, USA), and then centrifuged at 4200 rpm for 40 min at 4 °C. The GSH contents were measured at absorbance 412 nm using 2-nitrobenzoic acid (Sigma-Aldrich, St. Louise, MO, USA) as μM/mg tissue [47]. Decomposition of H_2_O_2_ in the presence of catalase was followed at 240 nm [48]. Catalase activity was defined as the amount of enzyme required to decompose 1 nM of H_2_O_2_ per minute, at 25 °C and pH 7.8. Results were expressed as U/mg tissue. The SOD activities were measured according to Sun *et al.* [49]. The SOD estimation was based on the generation of superoxide radicals produced by xanthine and xanthine oxidase, which react with nitrotetrazolium blue to form formazan dye. The activity was then measured at 560 nm by the degree of inhibition of this reaction, and was expressed as U/mg tissue.

### 2.13. Measurement of Hepatic Glucose-Regulating Enzyme Activities

The hepatic enzyme source was prepared as described elsewhere [50]. The hepatic tissue was homogenized in buffer solution (0.1 M triethanolamine, 0.2 M EDTA, and 0.002 M dithiothreitol) and centrifuged at 1000× *g* for 15 min at 4 °C. The supernatant was further centrifuged at 10,000× *g* for 15 min at 4 °C. The GK activity was measured based on the method of Davidson and Arion [51]. Briefly, reaction mixture (50 mM Hepes-NaGT (pH 7.4), 100 mM KCl, 7.5 mM MgCl_2_, 2.5 mM dithioerythritol, 10 mg/mL albumin, 10 mM glucose, 4 units of glucose-6-phosphate dehydrogenase, 50 mM NAD^+^, and 10 μL hepatic homogenates) was pre-incubated at 37 °C for 10 min. The reaction was initiated with the addition of 5 mM ATP and the mixture was further incubated at 37 °C for 10 min. The change in absorbance at 340 nm was recorded. The G6pase activity was measured based on method of Alegre *et al.* [52]. The reaction mixture contained 131.58 mM Hepes-NaGT (pH 6.5), 18 mM EDTA (pH 6.5), 265 mM glucose-6-phosphate, 10 μL of 0.2 M NADP^+^, 0.6 IU/mL mutarotase, and 0.6 IU/mL glucose dehydrogenase. After pre-incubation at 37 °C for 3 min, the mixture was added with sample homogenates and incubated at 37 °C for 4 min. The change in absorbance at 340 nm was measured. The PEPCK activity was measured using the method of Bentle and Lardy [53]. The reaction mixture contained 72.92 mM sodium Hepes (pH 7.0), 10 mM dithiothreitol, 500 mM NaHCO_3_, 10 mM MnCl_2_, 25 mM NADH, 100 mM IDP, 200 mM PEP, 7.2 unit of malic dehydrogenase, and 10 μL hepatic tissue homogenates. The enzyme activity was determined based on the decrease in the absorbance at 340 nm at 25 °C. All chemicals and reagents were obtained from Sigma-Aldrich (St. Louise, MO, USA).

### 2.14. Histopathology

Tissue samples were fixed in 10% neutral buffered formalin. The tissue were paraffin embedded, and serial-sectioned at 3–4 μm. The sections were stained with hematoxylin and eosin (H&E) for light microscopic examination. Additionally, the liver tissues were dehydrated in 30% sucrose, and cyro-sectioned for oil red stains [10,54]. For the histopathological changes, the steatohepatitis regions and hepatocyte diameters were measured using an automated image analysis process (*i*Solution FL v. 9.1, IMT *i*-solution Inc., Vancouver, QC, Canada) on the restricted view fields in H&E stain [10,14,15,54]. Steatohepatitis regions were calculated as percentages of fatty regions in the restricted view field of liver (%/mm^2^ of hepatic parenchyma) in oil red stain, and mean diameters of hepatocytes were also calculated on a computer monitor in H&E stain as μm of at least 10 hepatocytes per each view field. In addition, mean numbers of lipid droplet were also calculated among 100 renal tubules (number/100 tubules; at 1 field for sample), and mean diameters of white adipocytes in each fat fads were calculated in the restricted view fields as μm of long side of at least 10 white adipocytes per each fat pads. The thicknesses of fat pads (mm), mean areas occupied by zymogen granules (%/mm^2^ of pancreatic parenchyma), numbers of pancreatic islets (islets/10 mm^2^ of pancreatic parenchyma) and diameters of pancreatic islets (μm) were also measured, according to our previous methods [10,14,15]. The histopathologist was blinded to groups.

### 2.15. Immunohistochemistry

Serial sections of the pancreas were immunostained for insulin or glucagon using an avidin-biotin-peroxidase (ABC) methods [10]. Briefly, endogenous peroxidase activity was eliminated in methanol with 0.3% H_2_O_2_ for 30 min, and non-specific immunoglobulin was blocked with normal horse serum (Vector Lab., Burlingame, CA, USA. Dilution 1:100) for 1 h. The sections were treated with primary antiserum overnight at 4 °C in a humidity chamber; guinea pig polyclonal insulin (DiaSorin, Stillwater, MN, USA. Dilution: 1:2000) or rabbit polyclonal glucagon (DiaSorin, Stillwater, MN, USA. Dilution: 1:2000) antiserum. The next day, the sections were incubated with biotinylated universal secondary antibody (Vector Lab., Burlingame, CA, USA. Dilution 1:50) and ABC reagents (Vectastain Elite ABC Kit, Vector Lab., Burlingame, CA, USA. Dilution 1:50) for 1 h at room temperature. Finally, they were reacted with peroxidase substrate kit (Vector Lab., Burlingame, CA, USA) for 3 min at room temperature. All sections were rinsed in 0.01 M PBS 3 times, between steps. The cells occupied that more than 20% of immunoreactivities as compared with other naïve cells were regarded as positive. The immunopositive cells for insulin or glucagon were counted in the restricted pancreatic parenchyma (mm^2^) [10,55] and the ratios of cells were calculated using the following equation: Insulin/glucagon cells (ratio) = (mean numbers of insulin-immunoreactive cells/mean numbers of glucagon-immunoreactive cells). The histopathologist was blinded to the groups.

### 2.16. Statistical Analyses

All numerical values are expressed mean ± standard deviation (SD) of eight mice. Multiple comparison tests for different dose groups were conducted. Variance homogeneity was examined using the Levene test. If the Levene test indicated no significant deviations from variance homogeneity, the obtained data were analyzed by a one way ANOVA (Analysis of Variance) test followed by a least-significant differences multi-comparison (LSD) test to determine which pairs of group comparisons were significantly different. In case significant deviations from variance homogeneity were observed in the Levene test, a non-parametric comparison test, the Kruskal-Wallis H test, was conducted. When a significant difference was observed in the Kruskal-Wallis H test, the Mann-Whitney U (MW) test with Bonferroni correction was conducted to determine the specific pairs of group comparison that are significantly different. Statistical analyses were conducted using SPSS for Windows (Release 14.0K, IBM-SPSS Inc., Chicago, IL, USA). In addition, the percentage-point changes as compared with HFD control were calculated to help understand the efficacy of test substances, and the percentage-point changes between intact and HFD control were also calculated to observe disease inductions using the following equation: Percentage-point changes compared with intact control (%) = ( ((Data of HFD control − Data of intact control)/Data of intact control) × 100); Percentage-point changes compared with HFD control (%) = ( ((Data of test substance administered mice − Data of HFD control)/Data of HFD control) × 100), according to our previous report [10].

## 3. Results

### 3.1. Effects on Obesity

#### 3.1.1. Effects on the Body Weight Changes

**Figure 1:**
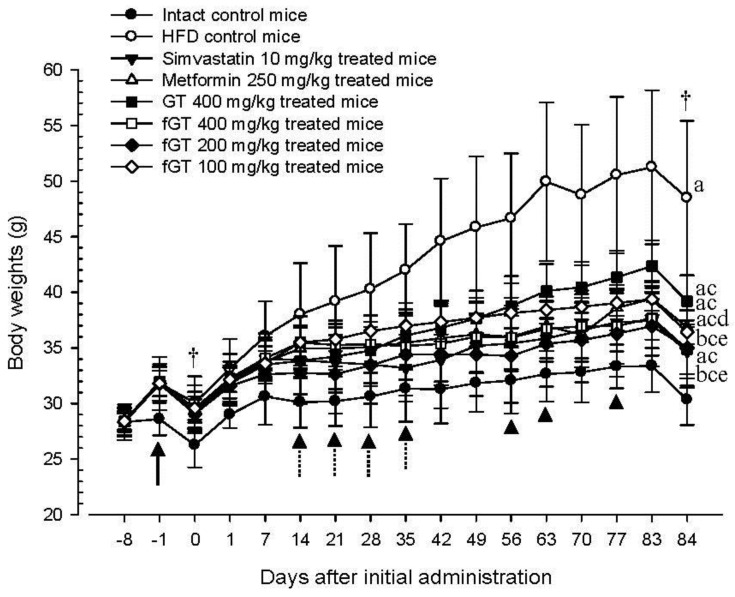
Body weight changes in NFD or HFD supplied mice. Values are expressed as Mean ± SD of eight mice. NFD, normal fat pellet diet; HFD, high fat diet; GT, green tea extracts; fGT, *Aquilariae lignum*-fermented green tea extracts. Simvastatin and metformin were administrated at dose levels of 10 and 250 mg/kg, respectively. All animals were overnight fasted before initial test substance administrations and sacrifice (†). ^a^ *p* < 0.01 and ^b^ *p* < 0.05 as compared with intact control; ^c^ *p* < 0.01 as compared with HFD control; ^d^ *p* < 0.01 and ^e^ *p* < 0.05 as compared with GT 400 mg/kg.

HFD control mice showed significant (*p* < 0.01) increases in body weight as compared with intact mice from 1 week after HFD supply (arrow), and accordingly, the body weight gains during 7 days of HFD adaption and 84 days of administration were also significantly (*p <* 0.01) larger as compared with the intact control. However, significant (*p <* 0.01 or *p <* 0.05) decreases of the body weights were detected in simvastatin 10 mg/kg, metformin 250 mg/kg, GT 400 mg/kg, fGT 400, 200 and 100 mg/kg treated mice from 14 to 35 days after start of administration as compared with HFD control (dot arrows), and accordingly, the body weight gains during 84 days of administration were also significantly (*p <* 0.01) lower in these groups as compared with HFD control, respectively. Especially, fGT 400, 200 and 100 mg/kg treated HFD mice also showed significant (*p <* 0.01 or *p <* 0.05) decreases of body weight from 63, 56 and 77 days after initial test substance administrations (arrowheads), and they also showed significantly (*p <* 0.01) lower body weight gains during the 84 days of continuous oral administration as compared with GT 400 mg/kg treated mice, respectively (Figure 1, Table 2).

The body weight gains during 84 days of administration in HFD control changed by 351 percentage points as compared with intact control, by −72%, −60%, −46%, −71%, −68% and −63% in simvastatin 10 mg/kg, metformin 250 mg/kg, GT 400 mg/kg, fGT 400, 200 and 100 mg/kg treated mice as compared with HFD control, respectively.

#### 3.1.2. Effects on Food Consumption and Energy Intake

Although significant (*p <* 0.01) decreases of mean daily food consumptions were detected in all HFD supplied mice as compared with intact control, no meaningful or significant changes on the mean daily food consumptions and the mean daily energy intakes were detected in all test substance administered groups including simvastatin 10 mg/kg as compared with HFD control, in this study. Energy intake per body weight was significantly decreased in groups of HFD control, metformin and GT 400 mg/kg, comparing to intact control. The decreases were related with increased body weights, but not with actual energy changes. Similarly, considering that there were significant decreases in the body weight gain in fGT 400 mg/kg treated group compared with HFD control, body weight gain was significantly decreased and there were no differences in daily food intake. (Table 2).

The mean daily food consumptions during 84 days of administration periods in HFD control were changed as −21% point as compared with intact control, but they were changed as 2%, −0%, 3%, 4%, 5% and 4% point in simvastatin 10 mg/kg, metformin 250 mg/kg, GT 400 mg/kg, fGT 400, 200 and 100 mg/kg treated mice as compared with HFD control, respectively. The mean daily energy intake per body weight during 84 days of administration periods in HFD control were changed as −32% point as compared with intact control, but they were changed as 26%, 19%, 20%, 25%, 32% and 21% point in simvastatin 10 mg/kg, metformin 250 mg/kg, GT 400 mg/kg, fGT 400, 200 and 100 mg/kg treated mice as compared with HFD control, respectively.

**Table 2:** Changes on body weight gains, mean daily food consumption and energy intake in NFD or HFD supplied mice.

Times Groups	Body Weight Gains (g) during		Mean Daily Food Consumption (g)	Mean Daily Energy Intake (kcal)	Mean Daily Energy Intake per Body Weight (kcal/g)
	Adapt Period	Administration Period			
Controls					
Intact	0.3 ± 0.2	4.1 ± 1.1	5.2 ± 0.7	21.0 ± 3.0	0.7 ± 0.1
HFD	3.5 ± 1.6 ^a^	18.4 ± 7.7 ^a^	4.1 ± 0.8 ^a^	19.5 ± 3.6	0.5 ± 0.1^a^
Reference					
Simvastatin	3.4 ± 1.4 ^a^	5.2 ± 2.2 ^c^	4.2 ± 0.9 ^a^	19.9 ± 4.0	0.6 ± 0.1
Metformin	3.5 ± 1.1 ^a^	7.4 ± 1.7 ^ac^	4.1 ± 0.9 ^a^	19.4 ± 4.5	0.5 ± 0.1^b^
GT 400 mg/kg	3.5 ± 1.8 ^a^	10.0 ± 0.9 ^ac^	4.3 ± 0.8 ^a^	20.1 ± 3.8	0.6 ± 0.1^b^
fGT treated					
400 mg/kg	3.5 ± 1.1 ^a^	5.3 ± 2.6 ^ce^	4.3 ± 0.8 ^a^	20.2 ± 3.8	0.6 ± 0.1^d^
200 mg/kg	3.5 ± 1.9 ^a^	5.9 ± 2.8 ^ce^	4.3 ± 0.7 ^a^	20.5 ± 3.5	0.6 ± 0.1
100 mg/kg	3.4 ± 1.6 ^a^	6.8 ± 0.9 ^ace^	4.3 ± 0.8 ^a^	20.3 ± 3.6	0.6 ± 0.1

Values are expressed as mean ± SD of eight mice. NFD, normal fat pellet diet; HFD, high fat diet; GT, green tea extracts; fGT, *Aquilariae lignum*-fermented green tea extracts. Simvastatin and metformin were administrated at dose levels of 10 and 250 mg/kg, respectively. All animals were overnight fasted. ^a^ *p* < 0.01 and ^b^ *p* < 0.05 as compared with intact control; ^c^ *p* < 0.01 and ^d^ *p* < 0.05 as compared with HFD control; ^e^ *p* < 0.01 as compared with GT 400 mg/kg.

#### 3.1.3. Effects on Body Fat Density: Total and Abdominal Fat Mass (%)

**Figure 2:**
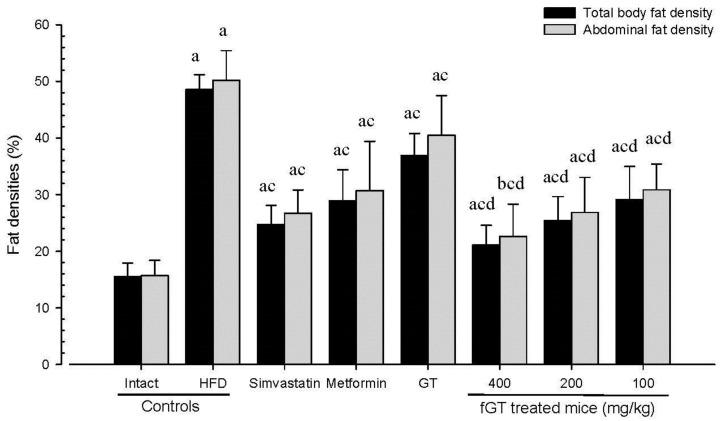
Total body and abdominal fat densities in NFD or HFD supplied mice. Values are expressed mean ± SD of eight mice. NFD, normal fat pellet diet; HFD, high fat diet; GT, green tea extracts; fGT, *Aquilariae lignum*-fermented green tea extracts; GT was administrated at a dose level of 400 mg/kg. Simvastatin and metformin were administrated at dose levels of 10 and 250 mg/kg, respectively. ^a^ *p* < 0.01 and ^b^ *p* < 0.05 as compared with intact control; ^c^ *p* < 0.01 as compared with HFD control; ^d^ *p* < 0.01 as compared with GT 400 mg/kg.

Significant (*p <* 0.01) increases of total body and abdominal fat densities were detected in HFD control as compared with intact control, respectively. On the contrary, a significant (*p <* 0.01) decrease of total body and abdominal fat masses were detected in all test substance treated mice including GT 400 mg/kg, during analysis of in live DEXA, respectively. Especially, fGT 400, 200 and 100 mg/kg treated HFD mice also showed significant (*p <* 0.01) decreases of total body and abdominal fat masses as compared with GT 400 mg/kg treated HFD mice, in this experiment (Figure 2).

The mean total body fat densities of HFD control were changed as 213.06% point as compared with intact control, but they were changed by −49%, −40%, −24%, −57%, −48% and −40% in simvastatin 10 mg/kg, metformin 250 mg/kg, GT 400 mg/kg, fGT 400, 200 and 100 mg/kg treated mice as compared with HFD control, respectively. The mean abdominal fat densities of HFD control changed by 220% as compared with the intact control, but they changed by −47%, −39%, −19%, −55%, −47% and −39% in simvastatin 10 mg/kg, metformin 250 mg/kg, GT 400 mg/kg, fGT 400, 200 and 100 mg/kg treated mice as compared with HFD control, respectively.

#### 3.1.4. Effects on the Periovarian and the Abdominal Wall Deposited Fat Pad Weights

Significant (*p <* 0.01) increases of periovarian deposited fat pad weights were detected in HFD control as compared with intact control. However, these increases of periovarian fat pad weights were significantly (*p <* 0.01) decreased by treatment of all test substances including fGT 400 mg/kg, in the both absolute and relative weights, respectively. Especially, all three different dosages of fGT treated HFD mice also showed significant (*p <* 0.01) decreases of the absolute and relative periovarian deposited fat pad weights as compared with GT 400 mg/kg treated HFD mice, respectively. Similar to those of periovarian deposited fat pads, significant (*p <* 0.01) increases of abdominal wall deposited fat pad absolute and relative weights were detected in HFD control as compared with intact control, respectively. However, these increases of abdominal wall deposited fat pad weights were significantly (*p <* 0.01) decreased by treatment of all test substances including metformin 250 mg/kg, in both absolute and relative weights, respectively. Especially, fGT 400, 200 and 100 mg/kg treated HFD mice also showed significant (*p <* 0.01) decreases of the absolute and relative abdominal wall deposited fat pad weights as compared with GT 400 mg/kg treated HFD mice, in this experiment (Table 3).

**Table 3:** Changes in relative organ weights (*% of body weights*) in NFD or HFD supplied mice.

Organs Groups	Liver	Kidney	Pancreas	Periovarian Fat Pads	Abdominal Wall Fat Pads
Controls					
Intact	3.8 ± 0.2	0.6 ± 0.1	0.6 ± 0.1	0.1 ± 0.0	0.2 ± 0.1
HFD	3.4 ± 0.4 ^a^	0.5 ± 0.1 ^a^	0.4 ± 0.1 ^a^	1.4 ± 0.3 ^a^	0.9 ± 0.2 ^a^
Reference					
Simvastatin	3.4 ± 0.3 ^b^	0.5 ± 0.0 ^b^	0.5 ± 0.1 ^bc^	0.3 ± 0.1 ^ac^	0.4 ± 0.1 ^ac^
Metformin	3.4 ± 0.3 ^b^	0.5 ± 0.1 ^b^	0.5 ± 0.0 ^ac^	0.4 ± 0.1 ^ac^	0.5 ± 0.1 ^ac^
GT 400 mg/kg	3.5 ± 0.3	0.5 ± 0.0	0.5 ± 0.1 ^ad^	0.9 ± 0.3 ^ac^	0.7 ± 0.2 ^a^
fGT treated					
400 mg/kg	3.3 ± 0.3 ^a^	0.5 ± 0.1 ^b^	0.6 ± 0.1 ^c^	0.3 ± 0.1 ^ace^	0.3 ± 0.1 ^ace^
200 mg/kg	3.4 ± 0.3 ^b^	0.5 ± 0.0	0.6 ± 0.1 ^c^	0.4 ± 0.1 ^ace^	0.4 ± 0.1 ^ace^
100 mg/kg	3.4 ± 0.3 ^b^	0.5 ± 0.0^b^	0.5 ± 0.1 ^bd^	0.5 ± 0.1 ^ace^	0.4 ± 0.2 ^ace^

Values are expressed as mean ± SD of eight mice. NFD, normal fat pellet diet; HFD, high fat diet; GT, green tea extracts; fGT, *Aquilariae lignum*-fermented green tea extracts. Simvastatin and metformin were administrated at dose levels of 10 and 250 mg/kg, respectively. ^a^ *p* < 0.01 and ^b^ *p* < 0.05 as compared with intact control; ^c^ *p* < 0.01 and ^d^ *p* < 0.05 and as compared with HFD control; ^e^ *p* < 0.01 as compared with GT 400 mg/kg.

The relative periovarian fat pad weights in HFD control changed by 1313.86% as compared with intact control, but they changed by −77%, −71%, −34%, −82%, −75% and −68% in simvastatin 10 mg/kg, metformin 250 mg/kg, GT 400 mg/kg, fGT 400, 200 and 100 mg/kg treated mice as compared with HFD control, respectively. The relative abdominal wall deposited fat pad weights in HFD control changed by 469% as compared with intact control, but they changed by −62%, −50%, −21%, −68%, −62% and −53% in simvastatin 10 mg/kg, metformin 250 mg/kg, GT 400 mg/kg, fGT 400, 200 and 100 mg/kg treated mice as compared with HFD control, respectively.

#### 3.1.5. Effects on the Adipocyte Histopathology in Periovarian and Abdominal Wall Deposited Fat Pads

**Figure 3:**
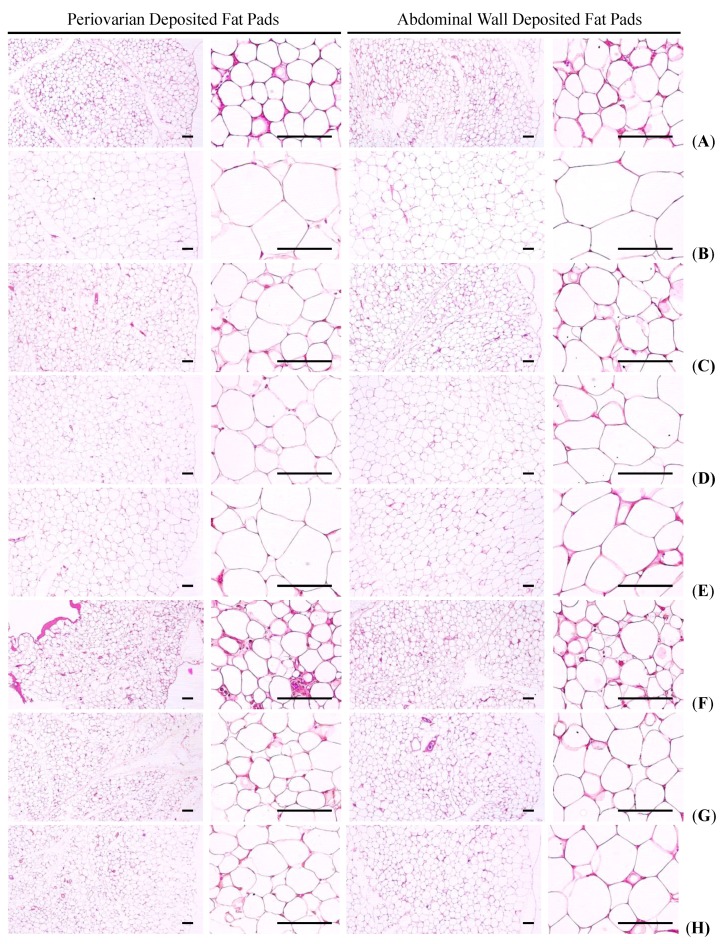
Representative histological images of the adipocytes, taken from NFD or HFD supplied mice periovarian and abdominal wall deposited fat pads. (**A**) Intact control: Normal pellet diet supplied vehicle control mice; 10 mL/kg of distilled water oral administered mice; (**B**) HFD (vehicle) control: 10 mL/kg of distilled water oral administered mice with HFD supply; (**C**) Simvastatin: 10 mg/kg of simvastatin oral administered mice with HFD supply; (**D**) Metformin: 250 mg/kg of metformin oral administered mice with HFD supply; (**E**) GT400: 400 mg/kg of GT oral administered mice with HFD supply; (**F**) fGT400: 400 mg/kg of fGT oral administered mice with HFD supply; (**G**) fGT200: 200 mg/kg of fGT oral administered mice with HFD supply; (**H**) fGT100: 100 mg/kg of fGT oral administered mice with HFD supply. NFD, normal fat pellet diet; HFD, high fat diet; GT, green tea extracts; fGT, *Aquilariae lignum*-fermented green tea extracts. All Hematoxylin & Eosin stain. Scale bars = 80 µm.

Significant (*p <* 0.01) increases of periovarian and abdominal white adipocyte diameters and thicknesses of each deposited fat pads were detected in HFD control as compared with intact control, respectively. However, these hypertrophy of adipocytes and fat depositions were significantly (*p <* 0.01) inhibited by treatment of all six test substances including metformin 250 mg/kg as compared with HFD control, respectively. Especially, fGT 400, 200 and 100 mg/kg treated HFD mice also showed significant (*p <* 0.01) decreases of the periovarian and abdominal wall deposited white adipocyte diameters and thicknesses of deposited each fat pads as compared with GT 400 mg/kg treated HFD mice, respectively (Table 4, Figure 3).

The deposited periovarian fat pad thicknesses in HFD control were changed as 255% point as compared with intact control, but they changed by −51%, −41%, −29%, −58%, −51% and −42% in simvastatin 10 mg/kg, metformin 250 mg/kg, GT 400 mg/kg, fGT 400, 200 and 100 mg/kg treated mice as compared with HFD control, respectively. The mean periovarian white adipocyte diameters in HFD control changed by 250% as compared with intact control, but they changed by −49%, −42%, −27%, −65%, −51% and −43% in simvastatin 10 mg/kg, metformin 250 mg/kg, GT 400 mg/kg, fGT 400, 200 and 100 mg/kg treated mice as compared with HFD control, respectively.

The abdominal wall deposited fat pad thicknesses in HFD control changed by 212% as compared with intact control, but they changed by −52%, −38%, −22%, −54%, −50% and −39% in simvastatin 10 mg/kg, metformin 250 mg/kg, GT 400 mg/kg, fGT 400, 200 and 100 mg/kg treated mice as compared with HFD control, respectively. The mean abdominal wall deposited fat pad white adipocyte diameters in HFD control changed by 180% as compared with intact control, but they changed by −43%, −38%, −27%, −54%, −48% and −40% in simvastatin 10 mg/kg, metformin 250 mg/kg, GT 400 mg/kg, fGT 400, 200 and 100 mg/kg treated mice as compared with HFD control, respectively.

**Table 4:** Changes in the histopathology-histomorphometry of the periovarian and abdominal wall deposited fat pads in NFD or HFD supplied mice.

Items Groups	Periovarian Fat Pads		Abdominal Wall Fat Pads	
	Thickness (mm)	Adipocyte Diameters (μm)	Thickness (mm)	Adipocyte Diameters (μm)
Controls				
Intact	1.5 ± 0.4	34.5 ± 12.2	1.9 ± 0.3	41.3 ± 10.7
HFD	5.2 ± 0.9 ^a^	120.5 ± 23.8 ^a^	5.8 ± 0.8 ^a^	115.6 ± 16.9 ^a^
Reference				
Simvastatin	2.6 ± 0.4 ^ab^	60.9 ± 10.9 ^ab^	2.8 ± 0.4 ^ab^	65.7 ± 12.9 ^ab^
Metformin	3.1 ± 0.5 ^ab^	70.1 ± 9.3 ^ab^	3.6 ± 0.9 ^ab^	71.1 ± 16.5 ^ab^
GT 400 mg/kg	3.7 ± 0.3 ^ab^	88.2 ± 12.0 ^ab^	4.5 ± 0.6 ^ab^	84.7 ± 13.7 ^ab^
fGT treated				
400 mg/kg	2.2 ± 0.5 ^abc^	42.2 ± 12.8 ^bc^	2.7 ± 0.7 ^abc^	53.1 ± 12.0 ^bc^
200 mg/kg	2.6 ± 0.5 ^abc^	58.7 ± 11.8 ^abc^	2.9 ± 0.5 ^abc^	60.2 ± 11.5 ^abc^
100 mg/kg	3.1 ± 0.2 ^abc^	69.0 ± 8.9 ^abc^	3.6 ± 0.5 ^abc^	69.4 ± 5.6 ^abd^

Values are expressed as mean ± SD of eight mice. NFD, normal fat pellet diet; HFD, high fat diet; GT, green tea extracts; fGT, *Aquilariae lignum*-fermented green tea extracts. Simvastatin and metformin were administrated at dose levels of 10 and 250 mg/kg, respectively. ^a^ *p* < 0.01 as compared with intact control; ^b^ *p* < 0.01 as compared with HFD control; ^c^ *p* < 0.01 and ^d^ *p* < 0.05 as compared with GT 400 mg/kg.

#### 3.1.6. Effects on the Exocrine Pancreas Zymogen Granule Contents

**Figure 4:**
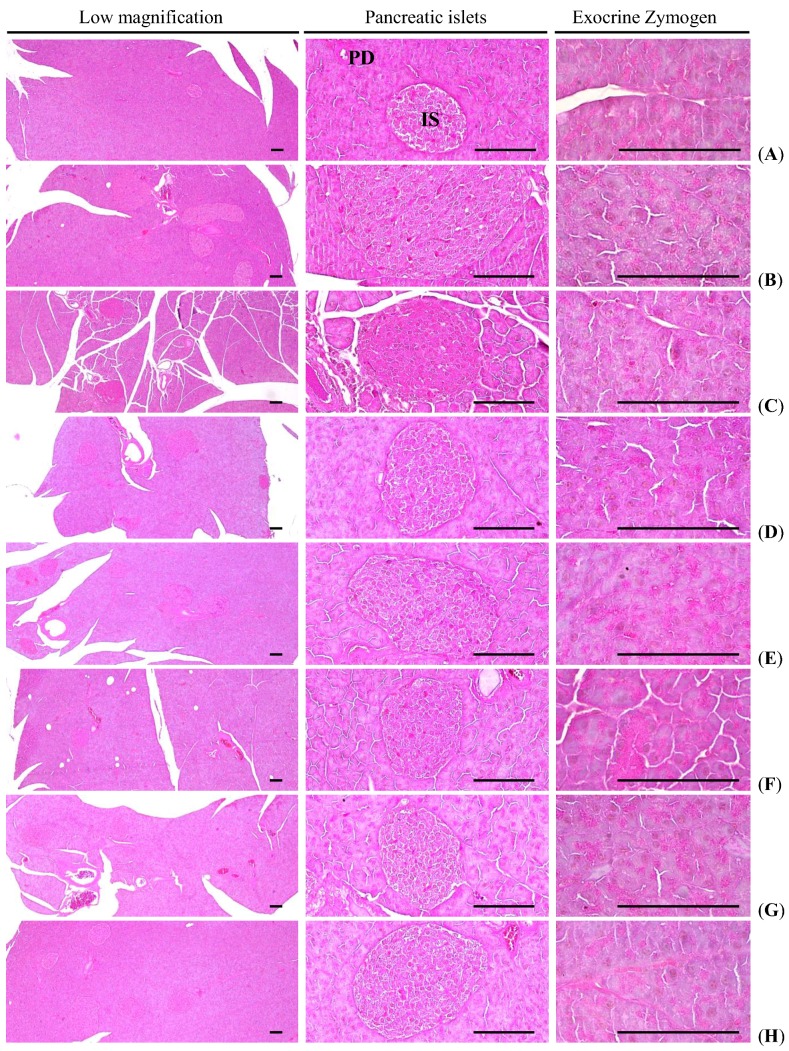
Representative general histological images of the pancreas, taken from NFD or HFD supplied mice. (**A**) Intact control: Normal pellet diet supplied vehicle control mice; 10 mL/kg of distilled water oral administered mice; (**B**) HFD (vehicle) control: 10 mL/kg of distilled water oral administered mice with HFD supply; (**C**) Simvastatin: 10 mg/kg of simvastatin oral administered mice with HFD supply; (**D**) Metformin: 250 mg/kg of metformin oral administered mice with HFD supply; (**E**) GT400: 400 mg/kg of GT oral administered mice with HFD supply; (**F**) fGT400: 400 mg/kg of fGT oral administered mice with HFD supply; (**G**) fGT200: 200 mg/kg of fGT oral administered mice with HFD supply; (**H**) fGT100: 100 mg/kg of fGT oral administered mice with HFD supply. NFD, normal fat pellet diet; HFD, high fat diet; GT, green tea extracts; fGT, *Aquilariae lignum*-fermented green tea extracts; IS, pancreatic islet; PD, pancreatic secretory duct. All Hematoxylin & Eosin stain. Scale bars = 80 µm.

Significant (*p <* 0.01) decreases in exocrine pancreas zymogen granule contents (the percentages of exocrine pancreas occupied by zymogen granules) were detected in HFD control as compared with intact control, as a result of the release of zymogen granules. However, exocrine pancreas zymogen granule contents were significantly (*p <* 0.01) increased in all test drug treated mice as compared with HFD control, except for simvastatin 10 mg/kg treated mice, in which the percentages of exocrine pancreas occupied by zymogen granules were similar to those of HFD control mice. Especially, all three different dosages of fGT treated HFD mice also showed significant (*p <* 0.01 or *p <* 0.05) increases of the percentage regions of exocrine pancreas occupied by zymogen granules as compared with GT 400 mg/kg treated HFD mice, in this experiment (Table 5, Figure 4).

The percentage regions of exocrine pancreas occupied by zymogen granule in HFD control were changed as −71% point as compared with intact control, but they changed by 15%, 179%, 102%, 294%, 217% and 175% in simvastatin 10 mg/kg, metformin 250 mg/kg, GT 400 mg/kg, fGT 400, 200 and 100 mg/kg treated mice as compared with HFD control, respectively.

**Table 5:** Changes in histopathology-histomorphometry of the pancreas in NFD or HFD supplied mice.

Items Groups	Zymogen Granules (%/mm^2^ of Exocrine)	Mean Islet Numbers (numbers/10 mm^2^)	Mean Islet Diameter (μm/islet)	Insulin-IR Cells (cells/mm^2^) [A]	Glucagon-IR Cells (cells/mm^2^) [B]	Insulin/Glucagon Ratio [A/B]
Controls						
Intact	50.1 ± 10.5	7.4 ± 2.5	95.5 ± 15.3	610.8 ± 110.6	168.3 ± 25.1	3.6 ± 0.2
HFD	14.3 ± 3.3 ^a^	30.1 ± 5.1 ^a^	289.9 ± 71.6 ^a^	2926.3 ± 197.4 ^a^	338.3 ± 21.4 ^a^	8.7 ± 0.3 ^a^
Reference						
Simvastatin	16.4 ± 4.9 ^a^	15.9 ± 3.5 ^ac^	150.3 ± 42.8 ^bc^	1155.6 ± 262.8 ^ac^	184.8 ± 42.5 ^c^	6.3 ± 0.7 ^ac^
Metformin	39.9 ± 11.4 ^bc^	17.1 ± 2.5 ^ac^	159.1 ± 22.4 ^ac^	1288.1 ± 217.6 ^ac^	207.8 ± 27.1 ^bc^	6.2 ± 0.9 ^ac^
GT 400 mg/kg	28.8 ± 2.6 ^ac^	22.6 ± 2.5 ^ac^	185.4 ± 21.7 ^ac^	2220.0 ± 365.9 ^ac^	294.8 ± 44.4 ^ac^	7.5 ± 0.5 ^ac^
fGT treated						
400 mg/kg	56.3 ± 10.9 ^cd^	12.4 ± 2.6 ^acd^	112.7 ± 17.3 ^cd^	875.9 ± 121.5 ^bcd^	175.3 ± 19.1 ^cd^	5.0 ± 0.8 ^acd^
200 mg/kg	45.3 ± 9.1 ^cd^	16.5 ± 2.8 ^acd^	148.1 ± 14.8 ^acd^	1104.8 ± 95.6 ^acd^	197.0 ± 21.5 ^cd^	5.7 ± 0.6 ^acd^
100 mg/kg	39.3 ± 8.8 ^bce^	17.9 ± 2.9 ^acd^	159.4 ± 11.6 ^ace^	1291.8 ± 157.3 ^acd^	219.9 ± 37.8 ^acd^	6.0 ± 0.7 ^acd^

Values are expressed as mean ± SD of eight mice. NFD, normal fat pellet diet; HFD, high fat diet; GT, green tea extracts; fGT, *Aquilariae lignum*-fermented green tea extracts; IR, immunoreactive. Simvastatin and metformin were administrated at dose levels of 10 and 250 mg/kg, respectively. ^a^ *p* < 0.01 and ^b^ *p* < 0.05 as compared with intact control; ^c^ *p* < 0.01 as compared with HFD control; ^d^ *p* < 0.01 and ^e^ *p* < 0.05 as compared with GT 400 mg/kg.

### 3.2. Anti-Diabetic Hypoglycemic Effects

#### 3.2.1. Effects on the Blood Glucose Levels

Significant (*p <* 0.01) increases in blood glucose levels were detected in HFD control as compared with intact control. However, the blood glucose levels were significantly (*p <* 0.01) reduced by treatment of all six test articles as compared with HFD control, except for simvastatin 10 mg/kg treated mice. Especially, fGT 400, 200 and 100 mg/kg treated HFD mice also showed significant (*p <* 0.01) decreases of the blood glucose levels as compared with GT 400 mg/kg treated HFD mice, respectively. Anyway, similar blood glucose levels were demonstrated in simvastatin 10 mg/kg treated mice as compared with those of HFD control mice, in this experiment (Table 6).

The blood glucose levels in HFD control changed by 215% as compared with intact control, but they changed by 10%, −45%, −30%, −59%, −48% and −45% in simvastatin 10 mg/kg, metformin 250 mg/kg, GT 400 mg/kg, fGT 400, 200 and 100 mg/kg treated mice as compared with HFD control, respectively.

#### 3.2.2. Effects on the Serum Insulin Levels

**Figure 5:**
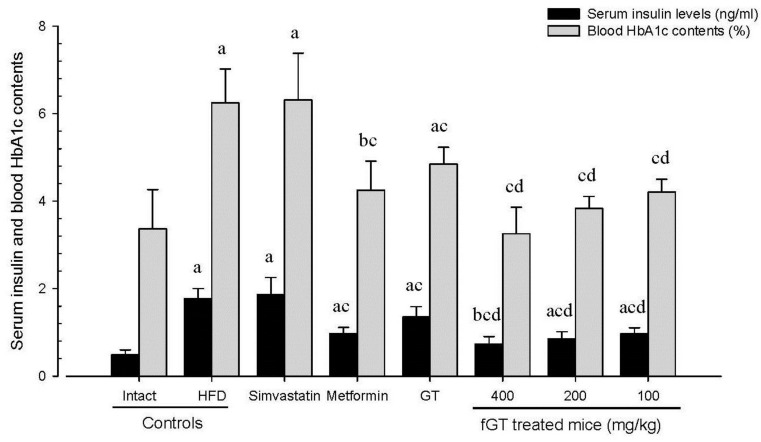
Serum insulin and blood HbA1c contents in NFD or HFD supplied mice. Values are expressed mean ± SD of eight mice. NFD, normal fat pellet diet; HFD, high fat diet; GT, green tea extracts; fGT, *Aquilariae lignum*-fermented green tea extracts; HbA1c, Glycated hemoglobin, hemoglobin A1c; GT was administrated at a dose level of 400 mg/kg. Simvastatin and metformin were administrated at dose levels of 10 and 250 mg/kg, respectively. ^a^ *p* < 0.01 and ^b^ *p* < 0.05 as compared with intact control; ^c^ *p* < 0.01 as compared with HFD control; ^d^ *p* < 0.01 as compared with GT 400 mg/kg.

Significant (*p <* 0.01) increases of serum insulin levels were detected in HFD control as compared with intact control. However, the serum insulin levels were significantly (*p <* 0.01) reduced by treatment of all six test articles as compared with HFD control except for simvastatin 10 mg/kg treated mice, in which similar serum insulin levels were demonstrated as compared with those of HFD control mice. Especially, all three different dosages of fGT 400, 200 and 100 mg/kg treated HFD mice also showed significant (*p <* 0.01) decreases of the serum insulin levels as compared with GT 400 mg/kg treated HFD mice, respectively (Figure 5). The serum insulin levels in HFD control changed by 265% as compared with intact control, but they changed by 5%, −45%, −24%, −59%, −52% and −45% in simvastatin 10 mg/kg, metformin 250 mg/kg, GT 400 mg/kg, fGT 400, 200 and 100 mg/kg treated mice as compared with HFD control, respectively.

**Table 6:** Changes in blood glucose levels and serum lipid contents in NFD or HFD supplied mice.

Items Groups	Glucose (mg/dL)	Total Cholesterol (mg/dL)	Triglyceride (mg/dL)	Low density Lipoprotein (mg/dL)	High Density Lipoprotein (mg/dL)
Controls					
Intact	97 ± 14	109 ± 20	46 ± 18	15 ± 2	100 ± 21
HFD	304 ± 64 ^a^	268 ± 26 ^b^	206 ± 16 ^a^	44 ± 10 ^a^	31 ± 12 ^a^
Reference					
Simvastatin	333 ± 40 ^a^	154 ± 43 ^bc^	112 ± 31 ^ac^	19 ± 3 ^ac^	85 ± 17 ^ac^
Metformin	168 ± 17 ^ac^	181 ± 23 ^ac^	130 ± 18 ^ac^	24 ± 4 ^ac^	67 ± 15 ^ac^
GT 400 mg/kg	212 ± 30 ^ac^	220 ± 31 ^ac^	160 ± 26 ^ac^	33 ± 4 ^ac^	52 ± 11 ^ac^
fGT treated					
400 mg/kg	126 ± 25^bc^^i^	134 ± 22 ^bcd^	85 ± 12 ^acd^	16 ± 3 ^cd^	93 ± 14 ^cd^
200 mg/kg	159 ± 31^ac^^i^	153 ± 16 ^acd^	113 ± 21 ^acd^	19 ± 2 ^acd^	85 ± 10 ^bcd^
100 mg/kg	169 ± 24^ac^^i^	177 ± 19 ^acd^	132 ± 17 ^acd^	24 ± 4 ^acd^	67 ± 8 ^ace^

Values are expressed as mean ± SD of eight mice. NFD = normal fat pellet diet; HFD, high fat diet; GT, green tea extracts; fGT, *Aquilariae lignum*-fermented green tea extracts. Simvastatin and metformin were administrated at dose levels of 10 and 250 mg/kg, respectively. ^a^ *p* < 0.01 and ^b^ *p* < 0.05 as compared with intact control; ^c^ *p* < 0.01 as compared with HFD control; ^d^ *p* < 0.01 and ^e^ *p* < 0.05 as compared with GT 400 mg/kg.

#### 3.2.3. Effects on the Blood HbA1c Contents

Significant (*p <* 0.01) increases of blood HbA1c contents were observed in HFD control as compared with intact control. However, the blood HbA1c contents were significantly (*p <* 0.01) reduced by treatment of all six test articles as compared with HFD control except for simvastatin 10 mg/kg treated mice, in which similar blood HbA1c contents were demonstrated as compared with those of HFD control mice. Especially, fGT 400, 200 and 100 mg/kg treated HFD mice also showed significant (*p <* 0.01) decreases of the blood HbA1c contents as compared with GT 400 mg/kg treated HFD mice, in this experiment (Figure 5).

#### 3.2.4. Effects on the Pancreatic Weights

Significant (*p <* 0.01) decreases of pancreas relative weights were detected in HFD control mice as compared with intact control mice. However, significant (*p <* 0.01 or *p <* 0.05) increases of pancreas relative eights were detected in simvastatin 10 mg/kg, metformin 250 mg/kg, GT 400 mg/kg, fGT 400, 200 and 100 mg/kg treated mice as compared with HFD control mice, in this experiment. Anyway, no meaningful changes in the absolute pancreatic weights were demonstrated in all experimental HFD mice including HFD control mice as compared with intact control, and also no significant changes on the absolute and relative weights were noticed in all three different dosages of fGT treated mice as compared with those of GT 400 mg/kg, in this study (Table 3). The relative pancreas weights in HFD control were changed as −37% point as compared with intact control, but they were changed as 33%, 24%, 20%, 41%, 36% and 29% point in simvastatin 10 mg/kg, metformin 250 mg/kg, GT 400 mg/kg, fGT 400, 200 and 100 mg/kg treated mice as compared with HFD control, respectively.

#### 3.2.5. Effects on the Pancreatic Islet Hyperplasia and Expansions

Significant (*p <* 0.01) increases of pancreatic islet numbers and mean diameters were detected in HFD control as compared with intact control, results from marked hyperplasia of pancreatic islet itself or component endocrine cells, respectively. However, these hyperplasia and expansion of islets were significantly (*p <* 0.01) reduced by treatment of all test substances including GT 400 mg/kg as compared with HFD control, respectively. Especially, fGT 400, 200 and 100 mg/kg treated HFD mice also showed significant (*p <* 0.01 or *p <* 0.05) decreases of the pancreatic islet numbers and mean diameters as compared with GT 400 mg/kg treated HFD mice, in this experiment (Table 5, Figure 4).

The mean pancreatic islet numbers in HFD control changed by 204% as compared with intact control, but they changed by −48%, −45%, −36%, −61%, −49% and −45% in simvastatin 10 mg/kg, metformin 250 mg/kg, GT 400 mg/kg, fGT 400, 200 and 100 mg/kg treated mice as compared with HFD control, respectively. The percentages of islet occupied regions in HFD control changed by 379% as compared with intact control, but they changed by −61%, −56%, −24%, −70%, −62% and −56% in simvastatin 10 mg/kg, metformin 250 mg/kg, GT 400 mg/kg, fGT 400, 200 and 100 mg/kg treated mice as compared with HFD control, respectively.

#### 3.2.6. Effects on the Pancreatic Islet Insulin- and Glucagon Cells

**Figure 6:**
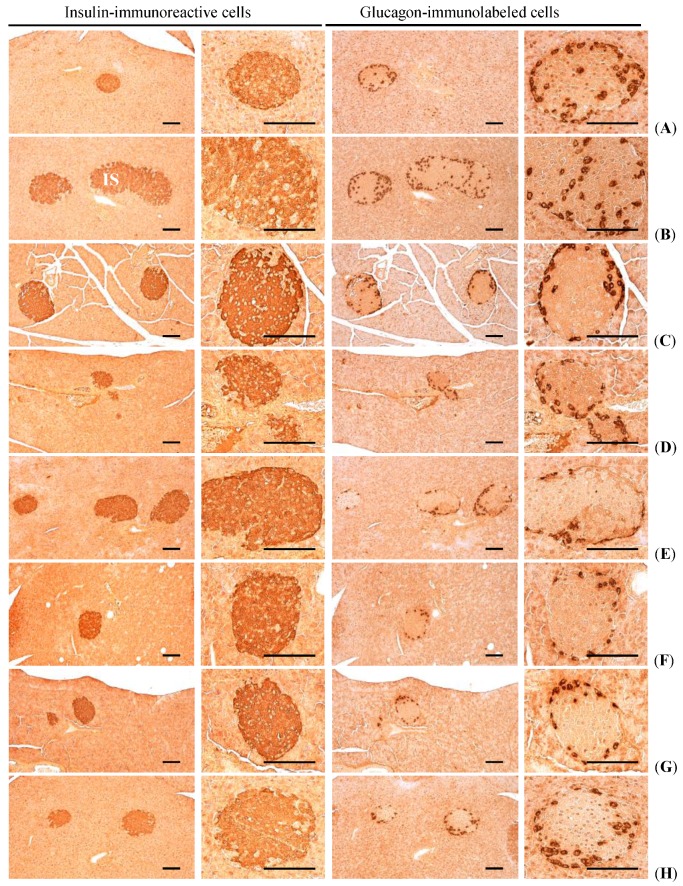
Representative histological images of the insulin- and glucagon-immunoreactive cells in the pancreas, taken from NFD or HFD supplied mice. (**A**) Intact control: Normal pellet diet supplied vehicle control mice; 10 mL/kg of distilled water oral administered mice; (**B**) HFD (vehicle) control: 10 mL/kg of distilled water oral administered mice with HFD supply; (**C**) Simvastatin: 10 mg/kg of simvastatin oral administered mice with HFD supply; (**D**) Metformin: 250 mg/kg of metformin oral administered mice with HFD supply; (**E**) GT400: 400 mg/kg of GT oral administered mice with HFD supply; (**F**) fGT400: 400 mg/kg of fGT oral administered mice with HFD supply; (**G**) fGT200: 200 mg/kg of fGT oral administered mice with HFD supply; (**H**) fGT100: 100 mg/kg of fGT oral administered mice with HFD supply. NFD, normal fat pellet diet; HFD, high fat diet; GT, green tea extracts; fGT, *Aquilariae lignum*-fermented green tea extracts. All immunostained by avidin-biotin-peroxidase complex. Scale bars = 80 µm.

Significant (*p* *<* 0.01) increases in insulin and glucagon-immunoreactive cells, and also insulin/glucagon cells were detected in HFD control mice as compared with intact control, respectively. However, these abnormal increases of insulin and glucagon-immunostained cells and their ratio (insulin/glucagon cells) were significantly (*p <* 0.01) normalized by treatment of all test substances including fGT 100 mg/kg as compared with HFD control, respectively. Especially, fGT 400, 200 and 100 mg/kg treated HFD mice also showed significant (*p <* 0.01) decreases of the insulin- and glucagon-immunolabeled cell numbers, insulin/glucagon cell ratios as compared with GT 400 mg/kg treated HFD mice, respectively (Table 5, Figure 6).

The mean numbers of insulin-immunoreactive cells in HFD control changed by 379% as compared with intact control, but they changed by −61%, −56%, −24%, −70%, −62% and −56% in simvastatin 10 mg/kg, metformin 250 mg/kg, GT 400 mg/kg, fGT 400, 200 and 100 mg/kg treated mice as compared with HFD control, respectively. The mean numbers of glucagon-immunolabeled cells in HFD control changed by 101% as compared with intact control, but they changed by −45%, −39%, −13%, −48%, −42% and −35% in simvastatin 10 mg/kg, metformin 250 mg/kg, GT 400 mg/kg, fGT 400, 200 and 100 mg/kg treated mice as compared with HFD control, respectively. The insulin/glucagon cells in HFD control changed by 139% point as compared with intact control, but they changed by −27%, −28%, −13%, −42%, −34% and −31% in simvastatin 10 mg/kg, metformin 250 mg/kg, GT 400 mg/kg, fGT 400, 200 and 100 mg/kg treated mice as compared with HFD control, respectively.

### 3.3. Effects on Hyperlipidemia

**Figure 7:**
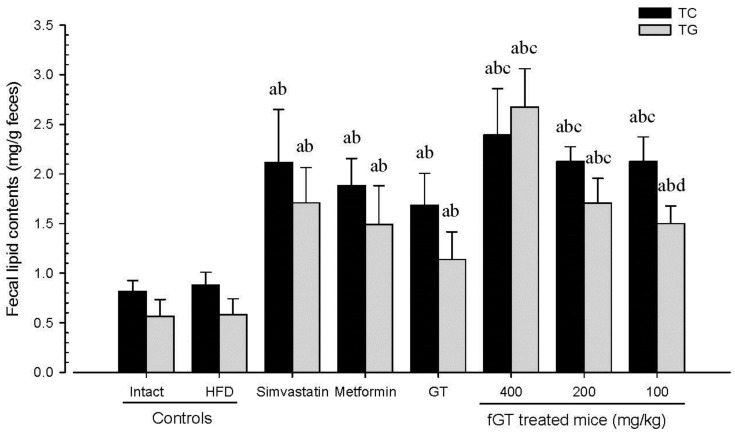
Fecal TC and TG content in NFD or HFD supplied mice. Values are expressed mean ± SD of eight mice. NFD, normal fat pellet diet; HFD, high fat diet; GT, green tea extracts; fGT, *Aquilariae lignum*-fermented green tea extracts; TC, total cholesterol; TG, triglyceride; GT was administrated at a dose level of 400 mg/kg. Simvastatin and metformin were administrated at dose levels of 10 and 250 mg/kg, respectively. ^a^ *p* < 0.01 as compared with intact control; ^b^ *p* < 0.01 as compared with HFD control; ^c^ *p* < 0.01 and ^d^ *p* < 0.05 as compared with GT 400 mg/kg.

A significant increases in serum TC, TG, and LDL levels and decreases in serum HDL levels were observed in HFD control as compared with intact control. In addition, all of the test substance-treated HFD mice showed significant decreases in serum TC, TG, and LDL levels, and increases in serum HDL levels compared with HFD control. Especially, fGT 400, 200 and 100 mg/kg treated HFD mice also showed significant changes of these indices compared with GT 400 mg/kg treated HFD mice (Table 6). Although slight, non-significant increases of fecal TC and TG contents were detected in HFD control as compared with intact control, the fecal TC and TG contents in all six test material treated mice including fGT 400 mg/kg were significantly elevated as compared with HFD control mice, respectively. Especially, fGT 400, 200 and 100 mg/kg treated HFD mice also showed significant increases of the fecal TC and TG contents as compared with GT 400 mg/kg treated HFD mice, in this experiment (Figure 7).

The serum TC levels in HFD control changed by 146% as compared with intact control, but they changed by −43%, −33%, −18%, −50%, −43% and −34% in simvastatin 10 mg/kg, metformin 250 mg/kg, GT 400 mg/kg, and fGT 400, 200 and 100 mg/kg treated mice as compared with HFD control, respectively. The serum TG levels in HFD control changed by 345% as compared with intact control, but they changed by −46%, −37%, −22%, −59%, −455 and −36% in simvastatin 10 mg/kg, metformin 250 mg/kg, GT 400 mg/kg, and fGT 400, 200 and 100 mg/kg treated mice as compared with HFD control, respectively. The serum LDL levels in HFD control changed by 203% as compared with intact control, but they changed by −57%, −46%, −27%, −65%, −57% and −46% in simvastatin 10 mg/kg, metformin 250 mg/kg, GT 400 mg/kg, and fGT 400, 200 and 100 mg/kg treated mice as compared with HFD control, respectively. The serum HDL levels in HFD control changed by −70% as compared with intact control, but they changed by 178%, 120%, 69%, 204%, 177% and 118% in simvastatin 10 mg/kg, metformin 250 mg/kg, GT 400 mg/kg, and fGT 400, 200 and 100 mg/kg treated mice as compared with HFD control, respectively.

The fecal TC contents in HFD control changed by 8% as compared with intact control, but they changed by 141%, 114%%, 92%, 172%, 142% and 142% in simvastatin 10 mg/kg, metformin 250 mg/kg, GT 400 mg/kg, and fGT 400, 200 and 100 mg/kg treated mice as compared with HFD control, respectively. The fecal TG contents in HFD control changed by 4% as compared with intact control, but they changed by 193%, 156%, 95%, 358%, 192% and 157% in simvastatin 10 mg/kg, metformin 250 mg/kg, GT 400 mg/kg, and fGT 400, 200 and 100 mg/kg treated mice as compared with HFD control, respectively.

### 3.4. Effects on Hepatopathy

#### 3.4.1. Effects on the Liver Weights

Significant (*p <* 0.01) increases of liver absolute weights were detected in HFD control as compared with intact control, respectively. However, these increases of absolute liver weights were significantly (*p <* 0.01) normalized by treatment of all six test substances including fGT 200 mg/kg treated mice as compared with HFD control mice, respectively. Especially, all three different dosages of fGT treated HFD mice also showed significant (*p <* 0.01) decreases of the liver absolute weights as compared with GT 400 mg/kg treated HFD mice, respectively. Although significant (*p <* 0.01) decreases of relative liver weights were also demonstrated in HFD control mice as compared with intact control mice, no significant changes in the relative liver weights were observed in all test substance administered mice as compared with HFD control mice, and fGT 400, 200 and 100 mg/kg treated mice did not show any significant changes in the relative liver weights as compared with those of GT 400 mg/kg treated mice, in this experiment (Table 3).

The relative liver weights in HFD control changed by −10% as compared with intact control, but they changed by 1%, 0%, 5%, −3%, 2% and −0% in simvastatin 10 mg/kg, metformin 250 mg/kg, GT 400 mg/kg, and fGT 400, 200 and 100 mg/kg treated mice as compared with HFD control, respectively.

#### 3.4.2. Effects on the Serum AST Levels

Significant (*p <* 0.01) increases of serum AST levels were detected in HFD control as compared with intact control. However, the serum AST levels were significantly (*p <* 0.01 or *p <* 0.05) decreased in all test substance administrated mice including fGT 100 mg/kg treated mice as compared with HFD control, respectively. Especially, fGT 400, 200 and 100 mg/kg treated HFD mice also showed significant (*p <* 0.01) decreases of the serum AST levels as compared with GT 400 mg/kg treated HFD mice, respectively (Table 7).

**Table 7:** Changes in serum AST, ALT, BUN and creatine levels in NFD or HFD supplied mice.

Items Groups	ALT (IU/L)	ALT (IU/L)	BUN (mg/dL)	Creatinine (mg/dL)
Controls				
Intact	71 ± 13	31 ± 11	31 ± 11	1 ± 0
HFD	218 ± 25 ^a^	165 ± 18 ^a^	91 ± 15 ^a^	2 ± 0 ^a^
Reference				
Simvastatin	116 ± 29 ^ac^	73 ± 20 ^ac^	54 ± 11 ^ac^	1 ± 0 ^ac^
Metformin	125 ± 25 ^ac^	85 ± 16 ^ac^	58 ± 11 ^ac^	1 ± 0 ^ac^
GT 400 mg/kg	183 ± 17 ^ad^	132 ± 15 ^ac^	73 ± 7 ^ac^	2 ± 0 ^ac^
fGT treated				
400 mg/kg	97 ± 16 ^ace^	63 ± 19 ^ac^^e^	41 ± 6 ^bc^^e^	1 ± 0 ^c^^e^
200 mg/kg	115 ± 13 ^ace^	74 ± 20 ^ac^^e^	51 ± 10 ^ac^^e^	1 ± 0 ^ac^^e^
100 mg/kg	126 ± 20 ^ace^	85 ± 14 ^ac^^e^	59 ± 10 ^ac^^f^	1 ± 0 ^ac^^f^

Values are expressed as Mean ± SD of eight mice. NFD, normal fat pellet diet; HFD, high fat diet; GT, green tea extracts; fGT, *Aquilariae lignum*-fermented green tea extracts; ALT, alanine aminotransferase; AST, aspartate aminotransferase; BUN, blood urea nitrogen. Simvastatin and metformin were administrated at dose levels of 10 and 250 mg/kg, respectively. ^a^ *p* < 0.01 and ^b^ *p* < 0.05 as compared with intact control; ^c^ *p* < 0.01 and ^d^ *p* < 0.05 as compared with HFD control; ^e^ *p* < 0.01 and ^f^ *p* < 0.05 as compared with GT 400 mg/kg.

The serum AST levels in HFD control changed by 205% as compared with intact control, but they changed by −47%, −43%, −16%, −55%, −47% and −42% in simvastatin 10 mg/kg, metformin 250 mg/kg, GT 400 mg/kg, and fGT 400, 200 and 100 mg/kg treated mice as compared with HFD control, respectively.

#### 3.4.3. Effects on the Serum ALT levels

Significant (*p <* 0.01) increases of serum ALT levels were detected in HFD control as compared with intact control. However, the serum ALT levels were significantly (*p <* 0.01) decreased in all test substance treated mice including simvastatin 10 mg/kg as compared with HFD control, respectively. Especially, all three different dosages of fGT 400, 200 and 100 mg/kg treated HFD mice also showed significant (*p <* 0.01) decreases of the serum ALT levels as compared with GT 400 mg/kg treated HFD mice, in this experiment (Table 7).

The serum ALT levels in HFD control changed by 438% as compared with intact control, but they changed by −56%, −49%, −20%, −62%, −55% and −49% in simvastatin 10 mg/kg, metformin 250 mg/kg, GT 400 mg/kg, and fGT 400, 200 and 100 mg/kg treated mice as compared with HFD control, respectively.

#### 3.4.4. Effects on the Steatohepatitis

**Figure 8:**
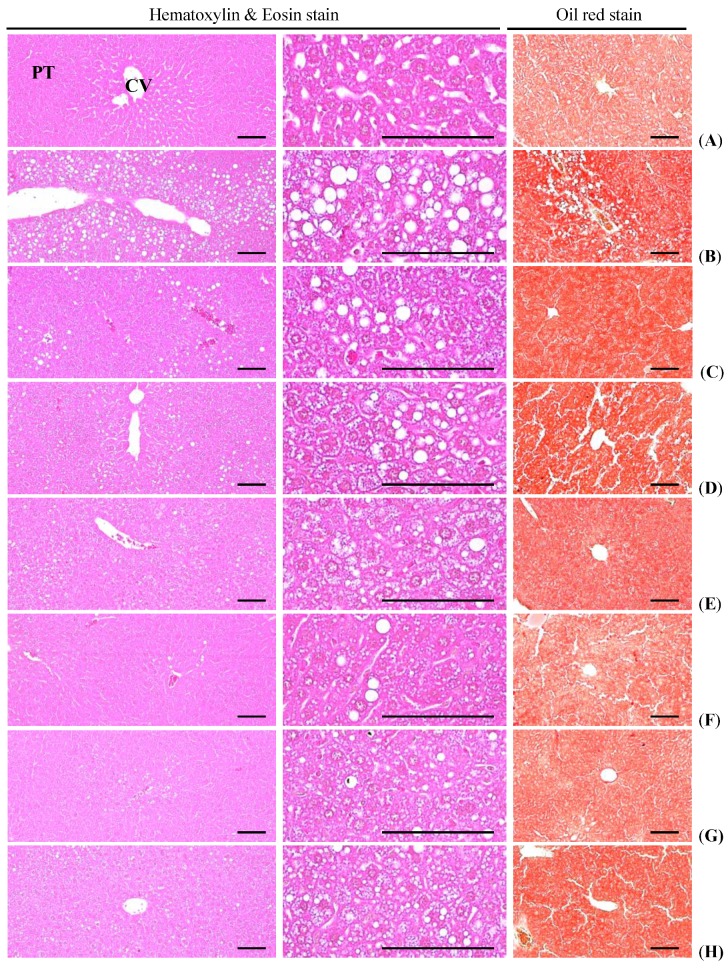
Representative histological images of the liver, taken from NFD or HFD supplied mice. (**A**) Intact control: Normal pellet diet supplied vehicle control mice; 10 mL/kg of distilled water oral administered mice; (**B**) HFD (vehicle) control: 10 mL/kg of distilled water oral administered mice with HFD supply; (**C**) Simvastatin: 10 mg/kg of simvastatin oral administered mice with HFD supply; (**D**) Metformin: 250 mg/kg of metformin oral administered mice with HFD supply; (**E**) GT400: 400 mg/kg of GT oral administered mice with HFD supply; (**F**) fGT400: 400 mg/kg of fGT oral administered mice with HFD supply; (**G**) fGT200: 200 mg/kg of fGT oral administered mice with HFD supply; (**H**) fGT100: 100 mg/kg of fGT oral administered mice with HFD supply. NFD, normal fat pellet diet; HFD, high fat diet; GT, green tea extracts; fGT, *Aquilariae lignum*-fermented green tea extracts; CV, central vein; PT, portal triad. Scale bars = 80 µm.

Significant (*p <* 0.01) increases of steatohepatitis (percentages of fatty changed regions in liver parenchyma) were detected in HFD control as compared with intact control, as a result of severe hypertrophy of hepatocyte related to intracellular lipid depositions. However, these steatohepatitis were significantly (*p <* 0.01) normalized by treatment of all five test substances including metformin 250 mg/kg treated mice, respectively. Especially, fGT 400, 200 and 100 mg/kg treated HFD mice also showed significant (*p <* 0.01) decreases of the steatohepatitis regions as compared with GT 400 mg/kg treated HFD mice, respectively (Table 8, Figure 8).

**Table 8:** Changes in histopathology-histomorphometry of the liver and kidney in NFD or HFD supplied mice.

Items Groups	Liver Steatosis (%/mm^2^ of Hepatic Tissues)	Mean Hepatocyte Diameters (μm/cell)	Degenerative Renal Tubule Numbers (%)
Controls			
Intact	7.1 ± 2.6	17.4 ± 1.4	2.6 ± 1.6
HFD	73.2 ± 10.7 ^a^	47.6 ± 10.2 ^a^	71.5 ± 12.3 ^a^
Reference			
Simvastatin	29.5 ± 10.1 ^ac^	24.2 ± 4.1 ^ac^	34.4 ± 14.4 ^ac^
Metformin	37.0 ± 13.7 ^ac^	27.8 ± 2.4 ^ac^	41.6 ± 14.0 ^ac^
GT 400 mg/kg	54.1 ± 12.2 ^ac^	34.7 ± 4.6 ^ac^	53.3 ± 10.3 ^ac^
fGT treated			
400 mg/kg	20.9 ± 10.3 ^bcd^	21.7 ± 3.5 ^acd^	7.6 ± 2.3 ^acd^
200 mg/kg	30.5 ± 10.6 ^acd^	24.1 ± 3.2 ^acd^	24.8 ± 8.0 ^acd^
100 mg/kg	36.7 ± 10.1 ^acd^	28.5 ± 2.6 ^acd^	35.3 ± 10.6 ^acd^

Values are expressed as Mean ± SD of eight mice. NFD, normal fat pellet diet; HFD, high fat diet; GT, green tea extracts; fGT, *Aquilariae lignum*-fermented green tea extracts. Simvastatin and metformin were administrated at dose levels of 10 and 250 mg/kg, respectively. ^a^ *p* < 0.01 and ^b^ *p* < 0.05 as compared with intact control; ^c^ *p* < 0.01 as compared with HFD control; ^d^ *p* < 0.01 as compared with GT 400 mg/kg.

The steatohepatitis regions in HFD control changed by 939% as compared with intact control, but they changed by −60%, −49%, −26%, −71%, −58% and −50% in simvastatin 10 mg/kg, metformin 250 mg/kg, GT 400 mg/kg, and fGT 400, 200 and 100 mg/kg treated mice as compared with HFD control, respectively.

#### 3.4.5. Effects on the Hepatocyte Hypertrophy

Significant (*p <* 0.01) increases of mean hepatocyte diameters (hypertrophy) were detected in HFD control as compared with intact control. However, these hepatocyte hypertrophies were markedly and significantly (*p <* 0.01) decreased in all six test substance treated mice including GT 400 mg/kg treated mice as compared with HFD control, respectively. Especially, all three different dosages of fGT treated HFD mice also showed significant (*p <* 0.01) decreases of the hepatocyte hypertrophies as compared with GT 400 mg/kg treated HFD mice, in this experiment (Table 8, Figure 8).

The mean hepatocyte diameters in HFD control changed by 173% as compared with intact control, but they changed by −49%, −42%, −27%, −54%, −49% and −40% in simvastatin 10 mg/kg, metformin 250 mg/kg, GT 400 mg/kg, fGT 400, 200 and 100 mg/kg treated mice as compared with HFD control, respectively.

### 3.5. Effects on Nephropathy

#### 3.5.1. Effects on the Kidney Weights

Significant (*p <* 0.01) increases of kidney absolute weights were detected in HFD control as compared with intact control, but they were significantly (*p <* 0.01) normalized by treatment of all six test materials including fGT 400 mg/kg as compared with HFD mice, in this study. Especially, fGT 400, 200 and 100 mg/kg treated HFD mice also showed significant (*p <* 0.01) decreases of the kidney absolute weights as compared with GT 400 mg/kg treated HFD control mice, respectively. Although significant (*p <* 0.01) decreases of kidney relative weights were demonstrated in HFD control mice as compared with intact NFD supplied control, no meaningful changes in the kidney relative weights were demonstrated in all test substance administrated mice as compared with HFD control mice, in this experiment. In addition, all three different dosages of fGT treated mice did not show any significant changes in the kidney relative weights as compared with those of GT 400 mg/kg treated mice, in the present study (Table 3).

The relative kidney weights in HFD control changed by −16% as compared with intact control, but they changed by 7%, 8%, 10%, 6%, 10% and 8% in simvastatin 10 mg/kg, metformin 250 mg/kg, GT 400 mg/kg, and fGT 400, 200 and 100 mg/kg treated mice as compared with HFD control, respectively.

#### 3.5.2. Effects on the Serum BUN Levels

Significant (*p <* 0.01) increases of serum BUN levels were detected in HFD control as compared with intact control. However, the serum BUN levels were significantly (*p <* 0.01) decreased in all six test substance treated HFD mice as compared with HFD control, respectively. Especially, all three different dosages of fGT treated HFD mice also showed significant (*p <* 0.01 or *p <* 0.05) decreases of the serum BUN levels as compared with GT 400 mg/kg treated HFD mice, in this experiment (Table 7).

The serum BUN levels in HFD control changed by 195% point as compared with intact control, but they changed −41%, −37%, −20%, −54%, −44% and −35% in simvastatin 10 mg/kg, metformin 250 mg/kg, GT 400 mg/kg, and fGT 400, 200 and 100 mg/kg treated mice as compared with HFD control, respectively.

#### 3.5.3. Effects on the Serum Creatinine Levels

Significant (*p <* 0.01) increases of serum creatinine levels were detected in HFD control as compared with intact control. However, the serum creatinine levels were significantly (*p <* 0.01) decreased in all test substance treated HFD mice including fGT 200 mg/kg treated mice as compared with HFD control mice, respectively. Especially, fGT 400, 200 and 100 mg/kg treated HFD mice also showed significant (*p <* 0.01 or *p <* 0.05) decreases of the serum creatinine levels as compared with GT 400 mg/kg treated HFD mice, respectively (Table 7).

The serum creatinine levels in HFD control changed by 229% as compared with intact control, but they changed −51%, −35%, −17%, −61%, −51% and −35% in simvastatin 10 mg/kg, metformin 250 mg/kg, GT 400 mg/kg, and fGT 400, 200 and 100 mg/kg treated mice as compared with HFD control, respectively.

#### 3.5.4. Effects on the Kidney Histopathology

**Figure 9:**
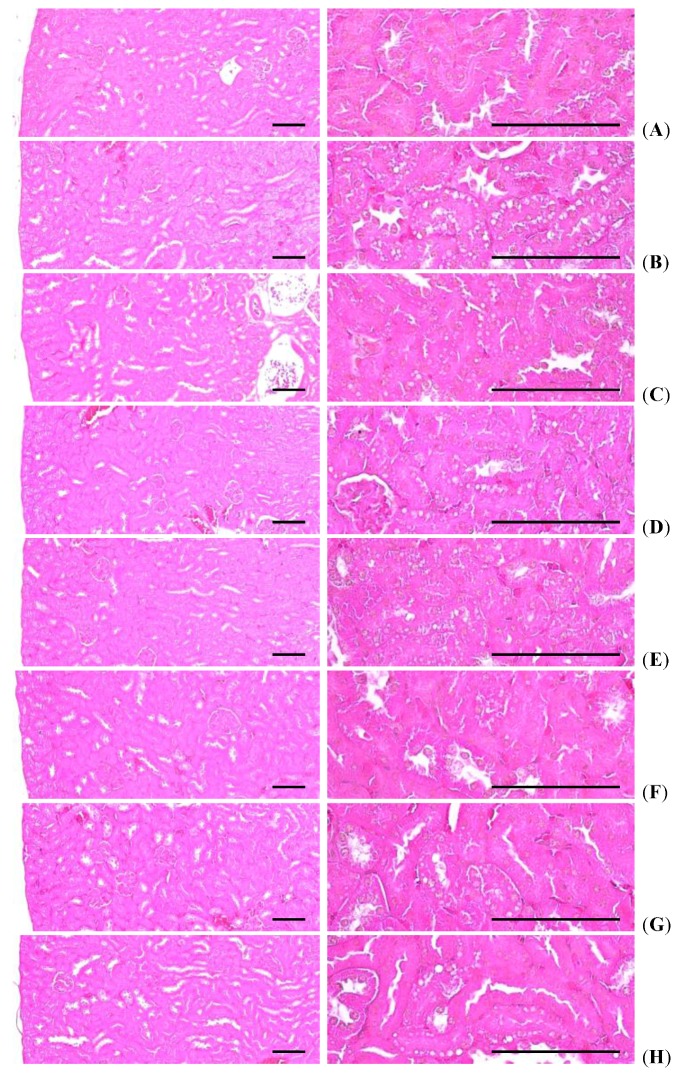
Representative histological images of the kidney, taken from NFD or HFD supplied mice. (**A**) Intact control: Normal pellet diet supplied vehicle control mice; 10 mL/kg of distilled water oral administered mice; (**B**) HFD (vehicle) control: 10 mL/kg of distilled water oral administered mice with HFD supply; (**C**) Simvastatin: 10 mg/kg of simvastatin oral administered mice with HFD supply; (**D**) Metformin: 250 mg/kg of metformin oral administered mice with HFD supply;(**E**) GT400: 400 mg/kg of GT oral administered mice with HFD supply; (**F**) fGT400: 400 mg/kg of fGT oral administered mice with HFD supply; (**G**) fGT200: 200 mg/kg of fGT oral administered mice with HFD supply; (**H**) fGT100: 100 mg/kg of fGT oral administered mice with HFD supply. NFD, normal fat pellet diet; HFD, high fat diet; GT, green tea extracts; fGT, *Aquilariae lignum*-fermented green tea extracts. All Hematoxylin & Eosin stain. Scale bars = 80 µm.

Significant (*p <* 0.01) increases of degenerative vacuolated renal tubules were detected in HFD control as compared with intact control, as a result of lipid droplets being deposited on diabetic nephropathies, but these diabetic nephropathies were significantly (*p <* 0.01) normalized by treatment of all six test materials including simvastatin 10 mg/kg treated mice as compared with HFD control, in our experiment. Especially, all three different dosages of fGT 400, 200 and 100 mg/kg treated HFD mice also showed significant (*p <* 0.01) decreases in the numbers of vacuolated renal tubules as compared with GT 400 mg/kg treated HFD mice, in this experiment (Table 8, Figure 9).

The numbers of degenerative vacuolated renal tubule in HFD control changed by 2624% as compared with intact control, but they changed by −52%, −42%, −26%, −89%, −65% and −51% in simvastatin 10 mg/kg, metformin 250 mg/kg, GT 400 mg/kg, and fGT 400, 200 and 100 mg/kg treated mice as compared with HFD control, respectively.

### 3.6. Effects on Liver Lipid Peroxidation and Antioxidant Defense System

#### 3.6.1. Effects on the Liver Lipid Peroxidation

Significant (*p <* 0.01) increases of liver lipid peroxidation and hepatic MDA content elevations were detected in HFD control as compared with intact control, but they were significantly (*p <* 0.01) normalized by treatment of all six test materials including fGT 100 mg/kg and simvastatin 10 mg/kg treated mice as compared with HFD control mice, respectively. Especially, fGT 400, 200 and 100 mg/kg treated HFD mice also showed significant (*p <* 0.01) decreases of the hepatic lipid peroxidation as compared with GT 400 mg/kg treated HFD mice, respectively (Table 9).

**Table 9:** Changes in the liver lipid peroxidation and antioxidant defense systems in NFD or HFD supplied mice.

Items Groups	Lipid Peroxidation	Antioxidant Defense System		
	Malondialdehyde (nM/mg Tissue)	Glutathione (μM/mg Tissue)	Catalase (U/mg Tissue)	SOD (U/mg Tissue)
Controls				
Intact	10.9 ± 1.7	33.5 ± 6.7	30.6 ± 9.1	2.8 ± 0.6
HFD	28.4 ± 3.4 ^a^	12.1 ± 1.9 ^a^	10.4 ± 2.6 ^a^	1.0 ± 0.1 ^a^
Reference				
Simvastatin	17.8 ± 2.2 ^a^^c^	19.8 ± 4.1 ^ac^	18.8 ± 1.8 ^ac^	2.0 ± 0.2 ^ac^
Metformin	20.1 ± 2.0 ^a^^c^	18.1 ± 1.9 ^ac^	17.5 ± 1.9 ^ac^	1.9 ± 0.2 ^ac^
GT 400 mg/kg	23.6 ± 2.7 ^a^^c^	16.5 ± 1.7 ^ac^	15.5 ± 1.5 ^ac^	1.4 ± 0.1 ^ac^
fGT treated				
400 mg/kg	15.2 ± 3.2 ^a^^cd^	26.4 ± 4.3 ^bcd^	22.1 ± 3.6 ^bcd^	2.3 ± 0.3 ^cd^
200 mg/kg	17.7 ± 1.7 ^a^^cd^	21.4 ± 2.4 ^acd^	19.1 ± 1.8 ^acd^	2.0 ± 0.1 ^acd^
100 mg/kg	20.1 ± 1.9 ^a^^cd^	18.8 ± 1.1 ^acd^	17.7 ± 1.3 ^ace^	1.9 ± 0.1 ^acd^

Values are expressed as mean ± SD of eight mice. NFD, normal fat pellet diet; HFD, high fat diet; GT, green tea extracts; fGT, *Aquilariae lignum*-fermented green tea extracts; SOD, superoxide dismutase. Simvastatin and metformin were administrated at dose levels of 10 and 250 mg/kg, respectively. ^a^ *p* < 0.01 and ^b^ *p* < 0.05 as compared with intact control; ^c^ *p* < 0.01 as compared with HFD control; ^d^ *p* < 0.01 and ^e^ *p* < 0.05 as compared with GT 400 mg/kg.

The hepatic lipid peroxidation in HFD control changed by 161% as compared with intact control, but they changed by −37%, −29%, −17%, −47%, −38% and −29% in simvastatin 10 mg/kg, metformin 250 mg/kg, GT 400 mg/kg, and fGT 400, 200 and 100 mg/kg treated mice as compared with HFD control, respectively.

#### 3.6.2. Effects on the Hepatic GSH Contents

Significant (*p <* 0.01) decreases of hepatic GSH—a representative endogenous antioxidant—contents were detected in HFD control as compared with intact control. However, the hepatic GSH contents were significantly (*p <* 0.01) increased in all test substance treated HFD mice including simvastatin 10 mg/kg treated mice as compared with HFD control mice, respectively. Especially, all three different dosages of fGT 400, 200 and 100 mg/kg treated HFD mice also showed significant (*p <* 0.01) increases of the hepatic GSH contents as compared with GT 400 mg/kg treated HFD mice, in this experiment (Table 9).

The hepatic GSH contents in HFD control changed by −64% as compared with intact control, but they changed by 63%, 50%, 36%, 119%, 77% and 56% in simvastatin 10 mg/kg, metformin 250 mg/kg, GT 400 mg/kg, and fGT 400, 200 and 100 mg/kg treated mice as compared with HFD control, respectively.

#### 3.6.3. Effects on the Hepatic CAT Activity

Significant (*p <* 0.01) decreases of hepatic CAT—a representative endogenous antioxidant enzyme—activities were detected in HFD control as compared with intact control, but these decreases of hepatic CAT activities were significantly (*p <* 0.01) normalized by treatment of all six test materials including metformin 250 mg/kg treated mice as compared with HFD control, in our experiment. Especially, fGT 400, 200 and 100 mg/kg treated HFD mice also showed significant (*p <* 0.01 or *p <* 0.05) increases of the hepatic CAT activities as compared with GT 400 mg/kg treated HFD mice, respectively (Table 9).

The hepatic CAT activities in HFD control changed by −66% point as compared with intact control, but they changed 81%, 68%, 49%, 112%, 83% and 70% in simvastatin 10 mg/kg, metformin 250 mg/kg, GT 400 mg/kg, and fGT 400, 200 and 100 mg/kg treated mice as compared with HFD control, respectively.

#### 3.6.4. Effects on the Hepatic SOD Activity

Significant (*p <* 0.01) decreases of hepatic SOD—another representative endogenous antioxidant enzyme—activities were detected in HFD control as compared with intact control, but they were significantly (*p <* 0.01) normalized by treatment of all six test materials including simvastatin 10 mg/kg treated mice as compared with HFD control mice, respectively. Especially, all three different dosages of fGT treated HFD mice also showed significant (*p <* 0.01) increases of the hepatic SOD activities as compared with GT 400 mg/kg treated HFD mice, in this experiment (Table 9).

The hepatic SOD activities in HFD control changed by −65% as compared with intact control, but they changed by 104%, 95%, 47%, 142%, 105% and 93% in simvastatin 10 mg/kg, metformin 250 mg/kg, GT 400 mg/kg, and fGT 400, 200 and 100 mg/kg treated mice as compared with HFD control, respectively.

### 3.7. Effects on Hepatic Glucose-Regulating Enzyme Activities

#### 3.7.1. Effects on the Hepatic GK Activity

Significant (*p <* 0.01) decreases of hepatic GK activities—a hepatic enzyme utilized in blood glucose—were detected in HFD control as compared with intact control, but they were significantly (*p <* 0.01 or *p <* 0.05) normalized by treatment of all six test materials as compared with HFD control mice, except for simvastatin 10 mg/kg treated mice in which similar hepatic tissue GK activities as HFD control were demonstrated in this experiment. Especially, fGT 400, 200 and 100 mg/kg treated HFD mice also showed significant (*p <* 0.01 or *p <* 0.05) increases of the hepatic GK activities as compared with GT 400 mg/kg treated HFD mice, respectively (Table 10).

The hepatic GK activities in HFD control changed by −29% as compared with intact control, but they changed by 4%, 26%, 15%, 35%, 34% and 28% in simvastatin 10 mg/kg, metformin 250 mg/kg, GT 400 mg/kg, fGT 400, 200 and 100 mg/kg treated mice as compared with HFD control, respectively.

#### 3.7.2. Effects on the Hepatic G6pase Activity

Significant (*p <* 0.01) increases of hepatic G6pase activities—a gluconeogenesis hepatic enzyme—were detected in HFD control as compared with intact control, but they were significantly (*p <* 0.01) normalized by treatment of all six test materials as compared with HFD control mice, except for simvastatin 10 mg/kg treated mice in this our experiment. Especially, all three different dosages of fGT treated HFD mice also showed significant (*p <* 0.01 or *p <* 0.05) decreases of the hepatic G6pase activities as compared with GT 400 mg/kg treated HFD mice, respectively. Anyway, simvastatin 10 mg/kg treated mice showed similar hepatic tissue GK activities as compared with HFD control, in this experiment (Table 10).

**Table 10:** Changes in the hepatic glucose-regulating enzyme activities in NFD or HFD supplied mice.

Items Groups	Glucokinase (nM/min/mg Protein)	Glucose-6-phosphatase (nM/min/mg Protein)	PEPCK (nM/min/mg Protein)
Controls			
Intact	2.8 ± 0.3	117.9 ± 12.8	2.0 ± 0.3
HFD	2.0 ± 0.2 ^a^	175.0 ± 14.3 ^a^	4.3 ± 0.6 ^a^
Reference			
Simvastatin	2.1 ± 0.3 ^a^	170.4 ± 22.6 ^a^	4.0 ± 1.0 ^a^
Metformin	2.5 ± 0.2 ^bc^	132.9 ± 11.6 ^bc^	2.7 ± 0.5 ^bc^
GT 400 mg/kg	2.3 ± 0.2 ^ad^	148.8 ± 12.3 ^ac^	3.5 ± 0.4 ^ac^
fGT treated			
400 mg/kg	2.7 ± 0.2 ^ce^	118.6 ± 11.1 ^ce^	2.2 ± 0.2 ^ce^
200 mg/kg	2.7 ± 0.2 ^ce^	126.4 ± 9.6 ^ce^	2.5 ± 0.4 ^bce^
100 mg/kg	2.5 ± 0.1 ^bcf^	133.0 ± 10.7 ^bcf^	2.7 ± 0.3 ^ace^

Values are expressed as mean ± SD of eight mice. NFD, normal fat pellet diet; HFD, high fat diet; GT, green tea extracts; fGT, *Aquilariae lignum*-fermented green tea extracts; PEPCK, Phosphoenolpyruvate carboxykinase. Simvastatin and metformin were administrated at dose levels of 10 and 250 mg/kg, respectively. ^a^ *p* < 0.01 and ^b^ *p* < 0.05 as compared with intact control; ^c^ *p* < 0.01 and ^d^ *p* < 0.05 as compared with HFD control; ^e^ *p* < 0.01 and ^f^ *p* < 0.05 as compared with GT 400 mg/kg.

The hepatic G6pase activities in HFD control changed by 49% as compared with intact control, but they changed by −3%, −24%, −15%, −32%, −28% and −24% in simvastatin 10 mg/kg, metformin 250 mg/kg, GT 400 mg/kg, and fGT 400, 200 and 100 mg/kg treated mice as compared with HFD control, respectively.

#### 3.7.3. Effects on the Hepatic PEPCK Activity

Significant (*p <* 0.01) increases of hepatic PEPCK activities, another gluconeogenesis hepatic enzyme, were detected in HFD control as compared with intact control, but they were significantly (*p <* 0.01) normalized by treatment of all six test materials as compared with HFD control mice, except for simvastatin 10 mg/kg treated mice in this our experiment. Especially, fGT 400, 200 and 100 mg/kg treated HFD mice also showed significant (*p <* 0.01) decreases of the hepatic PEPCK activities as compared with GT 400 mg/kg treated HFD mice, respectively. Anyway, simvastatin 10 mg/kg treated mice showed similar hepatic tissue PEPCK activities as compared with HFD control, in this experiment (Table 10).

The hepatic PEPCK activities in HFD control were changed as 113% point as compared with intact control, but they changed by −6%, −37%, −19%, −49%, −41% and −36% in simvastatin 10 mg/kg, metformin 250 mg/kg, GT 400 mg/kg, and fGT 400, 200 and 100 mg/kg treated mice as compared with HFD control, respectively.

## 4. Discussion

Diabetes mellitus is one of the primary threats to human health due to its increasing prevalence, chronic course, and disabling complications [6]. Since control of postprandial hyperglycemia and inhibition of oxidative stress are suggested to be important in the treatment of diabetes [6,10], many efforts had been made to search for effective and safe α-glucosidase inhibitors and antioxidants from natural materials to develop a physiological functional food or lead compounds for curing diabetes [7,8,10,14,15]. In our previous study [10], fGT also effectively inhibits diabetes and related complications—including diabetic hyperlipidemia, hepatopathies, nephropathies and obesities in db/db mice—more favorably than those of GT. In the present study, therefore, we intended to confirm or observe the real pharmacological activities of fGT in mildly diabetic obese mice, the HFD supplied mice [14,32,33,34] as compared with parent GT.

The increases of body mass and weights were meaningfully and dose-dependently inhibited by treatment of fGT and also by simvastatin, metformin and GT, respectively. The accumulation or increases of fat deposition in the body are major characteristics of obesity, and cellular hypertrophy appeared to be the major mode of expansion of the intra-abdominal adipose tissue in rodents [10,14,15]. In obesity, the increases in the accumulation of adipose tissues are common features in obesity. Adipose tissue is currently known to work not simply as an organ for energy storage, but also as an endocrine and secretory organ [56]. Adipose tissues secrete adipokines, and changes in the expression, secretion, and action of the adipokines in obesity are possibly implicated in the development of various diseases including insulin resistance [10,57]. In this study, treatment of fGT significantly inhibited the accumulation of adipose tissues and adipocyte hypertrophy, and especially, all three different dosages of fGT treated HFD mice also showed significant and more favorable inhibitory effects on adipose tissue accumulation and adipocyte hypertrophy as compared with those of GT.

The decreases of mean daily food consumption detected in all HFD supplied mice as compared with NFD supplied intact mice were not considered as critical problems in this study because the energy intake of HFD (4.73 kcal/g) used in the present study was much higher than that of normal diet (0.21 kcal/g). Similar decreases of daily food consumption in HFD supplied mice were already reported in our previous studies [10,15]. No meaningful or significant changes in the mean daily food consumptions were detected in all test substance administered groups as compared with HFD control, suggesting pharmacological effects of test substances detected in this study were difficult to associate with the inhibition of food consumption. It is generally known that obesity causes pancreatic steatosis, acinar cell atrophy, and a diminution in the number of zymogen granules [10,58]. The increases of zymogen granules in exocrine pancreatic acinar cells mean the production of digestive enzymes, especially for digestion of lipid and protein [59]. The diminishment of zymogen depositions in exocrine pancreas was effectively and dose-dependently inhibited by treatment of fGT and also by metformin but not in simvastatin. These results suggest that fermentation with appropriated amounts of *Aquilariae lignum*, 1:49 (2%; g/g), synergistically increased the anti-obese effects of GT in HFD mice, and may be mediated by inhibition of lipid digestions by decrease of pancreatic enzyme production or releases. Since it also could be completely excluded that fGT induced the increases of digestive tract motility, more detailed mechanism studies should be conducted in the future to elucidate the exact anti-obese mechanisms of fGT. Increased digestive tract motility also induced increases of fecal excretions, and, consequently, induced decreases of body weight [60,61,62] and dose-dependent, marked and noticeable increases of fecal excretions with fecal TC and TG contents were observed by treatment of fGT. The non-significant, slight elevation of fecal TC and TG contents detected in HFD control mice of this study is considered as a secondary result of HFD intake.

HbA1c is a form of hemoglobin that is measured primarily to identify the average plasma glucose concentration over prolonged periods of time, and produced by high glucose exposed erythrocytes for a long time [63]. Hyperglycemia is the main sign of diabetes, and hyperglycemia should be controlled to treat diabetes [10,14,15]. As progression of type II diabetes, marked elevations of blood insulin and Hb1Ac levels have been observed after long term HFD supply [34,41]. In addition, increased insulin secretion is in part related to pancreatic islet hyperplasia with progression of insulin-resistance by HFD supply [64,65,66]. Total pancreatic islet numbers and insulin-producing cells were increased after chronic consumption of a HFD, islets increased in area and number in order to secrete more insulin to try to maintain glucose homeostasis [67] with noticeable hypertrophy or hyperplasia in endocrine pancreas cells [64,65,66,67]. Also, in this study, noticeable elevations of blood glucose, insulin and HbA1c contents were detected in HFD control mice as compared with intact control, and with increases of pancreatic islet numbers, expansions, insulin- and glucagon-immunoreactive cells and insulin/glucagon cell ratios, suggesting the presence of type II diabetes in the histopathological observations. However, all three different dosages of fGT also effectively and dose-dependently inhibited these abnormal endocrine pancreas histopathological changes and elevations of blood glucose, insulin and HbA1c contents, respectively.

With the chronic development of diabetes in HFD mice, hyperlipidemia was also generally observed [6]. Since the most critical problem in hyperlipidemia is increases of serum LDL, TG and TC levels with a decrease of HDL levels [14,15,68], the efficacy of hypolipidemic agents was generally evaluated based on the decrease of serums LDL, TG and TC with an increase of HDL levels [10,14,15,69]. In this study, the treatment of fGT effectively and dose-dependently decreased the serum LDL, TG and TC levels, but favorably increased the serum HDL levels as compared with HFD control mice, suggesting fGT has favorable hypolipidemic effects on HFD mice. It considered that fermentation with *Aquilariae lignum*, synergistically increasing the hypolipidemic effects of GT, may be mediated by inhibition of lipid digestions by decrease of pancreatic enzyme production or releases. These hypolipidemic effects of test substances detected in these HFD mice were also considered as a result of the decreases of lipid absorptions and propulsion of lipids into feces, through the pancreatic digestive enzyme modulating effects as aforementioned.

With the progression of diabetes, increases of liver weight due to the fibrosis or abnormal glycosylation related hepatosteatosis and hepatocyte hypertrophic changes, due to lipid depositions in the cytoplasm, were observed with elevation of serum AST and ALT levels [10,15,70]. This phenomenon has been regarded as diabetic hepatopathy, and it also has been found in HFD supplied mice [15,71]. Improvement of these abnormal developments has been considered direct evidence of improved diabetic hepatopathies [70]. Also, the increases in kidney weights due to swelling, inflammation and necrotic processes were observed with elevation of serum BUN and creatinine levels—so-called diabetic nephropathy—and improvement of these abnormal developments has been considered direct evidence of improved diabetic nephropathies [10,15]. In this study, the treatment of fGT effectively and dose-dependently decreased the diabetic hepatopathies, inhibited the increases in liver weights, as well as serum AST and ALT elevations with steatohepatitis and related hepatocyte hypertrophic changes that occurred in histopathological observation, and the diabetic nephropathies, inhibited the increases in kidney weights, serum BUN and creatinine elevation with lipid droplet deposition related to renal tubule vacuolation in histopathological observation, as compared with HFD control mice, suggesting they have favorable hepatoprotective and nephroprotective effects on HFD mice.

There is considerable evidence of the role of free radicals in the etiology of diabetes and altered antioxidant defenses in diabetes [72]. Oxidative stress has been reported to play an important role in diabetes mellitus right from its genesis to the development of microvascular complications. Generation of free radicals by hyperglycemia is related to glucose auto-oxidation. Glucose auto-oxidation has been linked to non-enzymatic glycosylation, and glycosylated proteins have been shown to be a source of free radicals, ROS [10,73]. Oxidative stress in diabetes coexists with a decrease in the antioxidant status [74] which can increase the deleterious effects of free radicals. Generation of ROS related oxidative stress plays an important role in the etiology of diabetic complications [75]. Various toxic substances from lipid peroxidation destroy the surrounding tissues [76], and elevations of lipid peroxidation in the various organs were also demonstrated in HFD mice, and they also acted as a potent redox cycler that generates harmful ROS and causes organ damages [77,78]. GSH is representative of endogenous antioxidants and prevents tissue damage by keeping the ROS at low levels and at certain cellular concentrations, and is accepted as a protective endogenous antioxidant factor in tissues [79]. SOD is one of the antioxidant enzymes that contributes to enzymatic defense mechanisms, and CAT is an enzyme that catalyzes the conversion of H_2_O_2_ to H_2_O [80]. So, the increased lipid peroxidation and decrease of endogenous antioxidants—GSH contents, and antioxidant active enzymes—SOD and CAT activities in the damaged liver tissue are of secondary importance in terms of helping improve diabetes and various related complications [81,82]. In addition, marked elevation of hepatic lipid peroxidation, depletion of GSH content, and decreases of SOD and CAT activities were noticed in HFD control, like other previous HFD mice studies [41,83]. In this study, the treatment of fGT also effectively and dose-dependently inhibited the deterioration of the hepatic antioxidant defense system as compared with HFD control mice, suggesting favorable antioxidant effects of fGT on HFD mice.

The hepatic enzyme GK is related to glucose homeostasis and its increased expression could cause an increase in blood glucose utilization for energy production or glycogen storage in the liver, leading to a reduction in the blood glucose level [84]. On the contrary, the enzymes G6pase and PEPCK are associated with gluconeogenesis and hepatic glucose output and their increased activities denote increased glucose levels [85,86]. Generally, noticeable decreases of hepatic GK activities with increases of G6pase and PEPCK activities have been accompanied by HFD supply [41], and also in HFD control mice in this study. All three different dosages of fGT also effectively and dose-dependently inhibited HFD-induced hepatic glucose-regulating enzyme activity changes, the increases of hepatic GK activities, and decreases of hepatic G6pase and PEPCK activities as compared with those of HFD control mice.

## 5. Conclusions

The results obtained in this study suggest that fermentation with appropriated amounts of *Aquilariae lignum*, 1:49 (2%; g/g) synergistically increased the anti-diabetic effects of GT in HFD mice, through increases in the modulating effects on the hepatic glucose enzyme activities, the antioxidant and pancreatic lipid digestion enzymes, at least partially. All three different dosages of fGT treated mice showed dose-dependent favorable effects, and especially, fGT 100, 200 and 400 mg/kg treated HFD mice also showed significant and more favorable inhibitory activities against type II diabetes and related complications—including obesity, hyperlipidemia, hepatic steatosis, kidney failures and deterioration of the liver antioxidant defense system—as compared with those of GT 400 mg/kg treated mice, and also in hepatic GK, G6pase and PEPCK activities, respectively. Overall hypolipidemic effects fGT 200 mg/kg were similar to those of simvastatin 10 mg/kg, and fGT 100 mg/kg showed similar favorable effects on the HFD-induced diabetes as well as ameliorating effects on diabetic complications as compared with metformin 250 mg/kg, through enhancement of the liver antioxidant defense system, modulating effects on pancreatic digestive enzymes and hepatic glucose-regulating enzyme activities, at least partially. Accordingly, fGT is promising as a new potent therapeutic agent for type II diabetes and related complications, *i.e.*, metabolic syndrome.
